# Fault Tree Reliability Analysis via Squarefree Polynomials: Mathematical and Experimental Analysis

**DOI:** 10.1007/s42979-025-04450-y

**Published:** 2025-11-14

**Authors:** Milan Lopuhaä-Zwakenberg

**Affiliations:** Formal Methods and Tools, University of Twente, Enschede, the Netherlands

**Keywords:** Fault trees, Reliability analysis, Polynomial algebra

## Abstract

Quantitative analysis of risk models is essential to ensure the resilience of complex systems. Fault trees (FTs) form a ubiquitous prominent risk model, and unreliability is its key safety metric. As complex systems have larger and larger models, the complexity of algorithms computing unreliability is a pressing concern. Unfortunately, state-of-the-art algorithms, based on binary decision diagrams, do not give time complexity guarantees beyond a worst-case exponential bound. To address this issue, this paper introduces a new method to compute FT unreliability, extending the fast bottom-up algorithm for tree-shaped FTs to general FTs by framing its arithmetic in algebras of squarefree polynomials. We prove the validity of this algorithm, and that its time complexity is linear when the number of multiparent nodes is limited. Experiments establish the competitiveness of our new method.

## Introduction

*Fault trees* Fault tree (FT) analysis is a prominent risk assessment method to categorize safety risks on industrial systems. A FT is a hierarchical graphical model that describes how component failures propagate and lead to system failure. Because of its flexibility and rigour, FT analysis is incorporated in many risk assessment methods employed in industry, such as FaultTree+ [1] and TopEvent FTA [2].

**Fig. 1:**
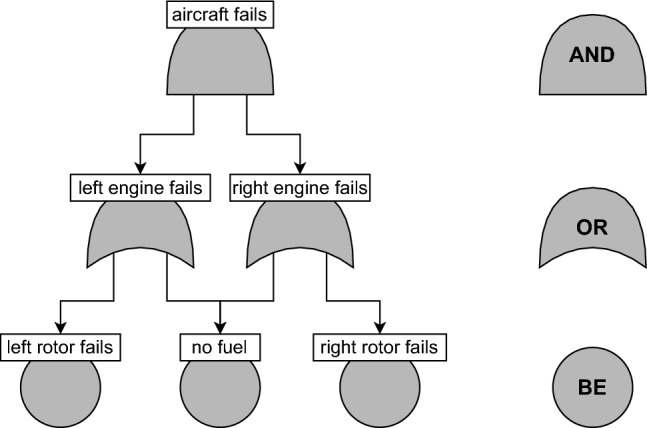
A fault tree for a small aircraft. The aircraft fails if both its engines fail; each engine fails if either its rotor fails or it has no fuel (the plane has a single fuel tank). Taken from [3, Fig. 1]

A FT is not necessarily a tree, but a directed acyclic graph whose root represents system failure. The leaves are called *basic events* (BEs) and represent atomic failure events. Intermediate nodes are AND/OR-gates, whose activation depends on that of their children. The system is considered to fail when the root is activated. An example is given in Fig. 1.

*Quantitative analysis*. Besides a qualitative analysis of what sets of events cause overall system failure, FTs also play an important role in *quantitative risk analysis*, which seeks to express the safety of the system in terms of safety metrics, such as the total expected downtime, availability, etc. An important safety metric is *(un)reliability*, which, given the failure probability of each BE, calculates the probability of system failure. Calculating the unreliability is crucial for giving safety and availability guarantees.

Two main approaches to calculating unreliability exist [4]. The first method works bottom-up, recursively calculating the failure probability of each gate. This algorithm is fast (linear time complexity), but only calculates unreliability correctly when the FT is actually a tree. However, this is rare in practice: nodes with multiple parents (DAG-like FTs) are necessary to model more intricate systems. For such FTs, as shown in this paper, calculating unreliability is NP-hard.

The second method for unreliability calculation is based on translating the FT into a binary decision diagram (BDD) and performing a bottom-up analysis on the BDD. This BDD is of worst-case exponential size, though heuristics exist. The BDD corresponding to an FT depends on choosing a linear ordering of the BEs, and different orderings yield BDDs of wildly varying size. Although any BDD can be used to calculate unreliability, finding the optimal BE ordering is an NP-hard problem in itself. Hence it is hard to give guarantees on the time complexity of calculating unreliability via BDDs, in terms of properties of the FT.

*Contributions.* This paper presents a radical new way for calculating unreliability for general FTs. The bottom-up algorithm does not work for DAG-like FTs, since it does not recognize multiple copies of the same node in the calculation, leading to double counting. Our approach amends this by keeping track of nodes with multiple parents, as these may occur twice in the same calculation. Then, instead of propagating failure probabilities as real numbers, we propagate squarefree polynomials whose variables represent the failure probabilities of nodes with multiple parents. Keeping these multiparent nodes as formal variables allows us to detect and account for double counting. During this propagation, to keep complexity down, formal variables are replaced with real numbers as soon as possible to ensure that the size of the polynomials remains small. Arriving at the root, all formal variables have been substituted away, yielding the unreliability as a real number.

Unlike BDD-based algorithms, this allows us to give upper bounds to computational complexity. Most of the complexity comes from the fact that we do arithmetic with polynomials rather than real numbers. However, if the number of multiparent nodes is limited, these polynomials have limited degree, and computation is still fast. We prove this formally, by showing that the time complexity is linear when the number of multiparent nodes is bounded. We also compare the performance to STORM [5], a state-of-the-art FT analysis tool using BDDs, and Coyan [6], a novel FT analysis tool based on weighted model counts. We find that time-wise, our method is competitive with STORM and is outperformed by Coyan; memory-wise, our method outperforms STORM and is competitive with Coyan.

Summarized our contributions are the following:

1. A new algorithm for fault tree reliability analysis based on squarefree polynomial algebras;
2. A proof of the algorithm’s validity and bounds on its time complexity;
3. Experiments comparing our algorithm to the state-of-the-art.

This paper is an extended version of [3], presented at MODELSWARD 2024. Compared to that version, this paper has the following contributions:

1. A more elaborate discussion of the state of the art, including new developments since the publication of [3];
2. Proofs and detailed examples of the mathematical results;
3. A more elaborate experimental section with more benchmarks including real-world FTs, and comparison to Coyan [6].

The artifact of the experiments is available at [7].

## Related Work

Owing to its relevance to risk management, FT analysis has received considerable attention in the literature. FTs were first introduced in [8]. Qualitative fault tree analysis mostly centers on finding *minimal cut sets*, i.e., sets of failure events that together affect the system’s functioning [9, 10]. Quantitative FT analysis is mostly concerned with reliability, i.e., the probability of system failure within its mission time. A bottom-up algorithm to calculate their reliability is presented in [4], formalizing principles traditionally used in FT analysis. This algorithm only computes reliability correctly for FTs that are actually trees, i.e., do not contain nodes with multiple parents. A novel method for calculating reliability that also works for general, DAG-shaped FTs was introduced in [11]. This approach translates the Boolean function underlying the FT into a binary decision diagram (BDD). A bottom-up algorithm on the BDD then computes the unreliability. While the BDD is worst-case of exponential size, this is usually quite fast in practice.

Since its inception, two major improvements have been introduced to the BDD method. The first one is heuristics for variable order: BDD size is heavily dependent on variable order, but finding the optimal variable order is NP-hard by itself. Heuristics are therefore a useful tool in reducing BDD size [12]. The second improvement is *modular decomposition*: when a sub-FT is only connected to the larger FT through its root, it can be split off and have its reliability handled separately, giving rise to a more efficient divide-and-conquer approach [13]. Unfortunately, this approach is very sensitive to the FT structure: if there is even one additional edge present between the sub-FT and the larger FT, this decomposition can no longer be applied and the full BDD needs to be constructed. Nevertheless, the BDD method with variable reordering heuristics and modular decomposition is the de facto standard for FT reliability analysis, being implemented in all state-of-the-art tools such as XFTA [14], SCRAM [15] and STORM [16].

Recently, a third method method was proposed based on weighted model count (WMC) [6]. The key insight is that with appropriately chosen weights, unreliability is the weighted model count of the Boolean formula that the FT represents. After finding a suitable translation of this Boolean formula into conjunctive normal form, [6] uses state-of-the-art WMC solvers [17, 18] to find FT unreliability. These solvers still underlyingly use BDDs; nevertheless, using WMC results in a significant increase in performance. This approach is somewhat related to the work [19], where SAT solving is used to evaluate Boolean queries on FTs; this approach, however, has not yet been generalized to probabilistic queries [20], of which FT unreliability is the most example.

A number of extensions and variations of the FT formalism exist. The most prominent one are dynamic fault trees (DFTs) [21], which assign to each basic event a failure time as a continuous random variable; DFT analysis focuses on the development of reliability over time. Furthermore, DFTs have extra gate types, such as SPARE gates, that model time-related behaviour. DFTs are analyzed by translating them into stochastic transition systems, such as continuous-time Markov chains [22], Petri nets [23] or Bayesian networks [24]; for an overview see [25]. the key challenge in DFT analysis is to prevent state space explosion; for an overview of techniques see [26].

Another variant of FTs are attack trees (ATs) [27]; these are mathematically similar to FTs but model security rather than safety. Rather than reliability, AT analysis considers a variety of metrics such as attacker skill, attack time and attack cost. At analysis works by creating a common algebraic framework for these methods based on semirings, and developing algorithms in this general framework [28–30]. ATs themselves also have many extensions, such as dynamic attack trees [31], attack-defense trees [32] and attack-fault trees [33], each with their own types of analysis.

Finally, FTs can be considered a special type of Bayesian networks with deterministic inner nodes, and FT reliability becomes Bayesian inference under this identification; for an overview of inference algorithms see [34]. Such methods are applied for FT reliability in [35].

## Fault Trees

In this section, we give the formal definition of fault trees (FTs) and their reliability, as is used in this paper. For us FTs are static (only AND/OR gates), and each basic event *v* is assigned a failure probability $\textrm{p}(v)$. Thus we us the following definition:

### Definition 1

A *fault tree* (FT) is a tuple $T = (V,E,\gamma ,\textrm{p})$ where:

- ($V$, *E*) is a rooted directed acyclic graph;
- $\gamma$ is a function $\gamma\! :V \rightarrow \{\mathtt{OR},\mathtt{AND},\mathtt{BE}\}$ such that $\gamma (v) = \texttt{BE}$ if and only if $v$ is a leaf;
- $\textrm{p}$ is a function $\textrm{p}\!:\operatorname {BE}_{T} \rightarrow [0,1]$, where $\operatorname {BE}_{T} = \{v \in V \mid \gamma (v) = \texttt{BE}\}$.

Despite its terminology, that a FT is not necessarily a tree in the graph-theoretic sense, as gates may share children. The root of $T$ is denoted $\operatorname {R}_{T}$. For a node $v$, we let $\operatorname {ch}(v)$ be the set of children of $v$.

The *structure function* determines, given a gate and a safety event, whether the event successfully propagates to the gate. Here we model a safety event as the set of BEs happening, which can be encoded as a binary vector $\vec {\sigma } \in {\mathbb {B}}^{\operatorname {BE}_{T}}$, where $\sigma _v = \texttt{1}$ denotes that the BE *v* occurs in the event. The structure function is then defined as follows.

### Definition 2

Let $T = (V,E,\gamma ,\textrm{p})$ be a FT.

1. A *safety event* is an element of ${\mathbb {B}}^{\operatorname {BE}_{T}}$.
2. The *structure function* of $T$ is the function $\operatorname {S}_{T}:V \times {\mathbb {B}}^{\operatorname {BE}_{T}} \rightarrow {\mathbb {B}}$ defined recursively by
  $$ \operatorname {S}_{T}(v,\vec {\sigma }) = {\left\{ \begin{array}{ll} \sigma _v, & \text { if } \gamma (v) = \texttt{BE}\text {,}\\ \bigvee _{w \in \operatorname {ch}(v)} \operatorname {S}_{T}(w,\vec {\sigma }), & \text { if } \gamma (v) = \texttt{OR},\\ \bigwedge _{w \in \operatorname {ch}(v)} \operatorname {S}_{T}(w,\vec {\sigma }), & \text { if } \gamma (v) = \texttt{AND}. \end{array}\right. } $$
3. A safety event $\vec {\sigma }$ such that $\operatorname {S}_{T}(\operatorname {R}_{T},\vec {\sigma }) = \texttt{1}$ is called a *cut set*; the set of all cut sets is denoted $\operatorname {CS}_{T}$.

Quantitative analysis of a FT is done via its *unreliability*, i.e., the probability of a cut set occurring, where each BE $v$ has probability $\textrm{p}(v)$ of happening. The BE failure probabilities are considered to be independent. This is because when they are not independent, this is due to some common cause; this common cause should then be explicitly modeled in the FT framework, by replacing non-independent BEs with sub-FTs with common nodes [36].

### Definition 3

Let $T = (V,E,\gamma ,\textrm{p})$ be a FT. Let $\vec {\Sigma } \in {\mathbb {B}}^{{\operatorname {BE}_{T}}}$ be the random variable defined by $\mathbb {P}(\Sigma _v = \texttt{1}) = \textrm{p}(v)$ for all $v \in \operatorname {BE}_{T}$, and all these events are independent. Then the *unreliability* of *T* is defined as

$$\begin{aligned} U(T)&= \mathbb {P}(\vec {\Sigma } \in \operatorname {CS}_{T}) \\ &= \sum _{\vec {\sigma } \in \operatorname {CS}_{T}} \prod _{v:\sigma _v = \texttt{1}} \textrm{p}(v)\prod _{v:\sigma _v = \texttt{0}} (1-\textrm{p}(v)). \end{aligned}$$

### Example 4

Consider the FT $T$ from Fig. 1. Abbreviating BE names, assume $\textrm{p}(\mathsf{rrf}) = \textrm{p}(\mathsf{lrf}) = 0.4$ and $\textrm{p}(\mathsf{nf}) = 0.3$. Furthermore, write $\vec {\sigma } \in {\mathbb {B}}^{\operatorname {BE}_{T}}$ as $\sigma _{\mathsf{rrf}}\sigma _{\mathsf{nf}}\sigma _{\mathsf{lrf}}$. Then $\operatorname {CS}_{T} = \{010,011,101,110,111\}$, so

$$\begin{aligned} U(T)&= 0.6 \cdot 0.3 \cdot 0.6 + 0.6 \cdot 0.3 \cdot 0.4 + 0.4 \cdot 0.7 \cdot 0.4 \\ &\quad + 0.4 \cdot 0.3 \cdot 0.6 + 0.4 \cdot 0.3 \cdot 0.4 = 0.412. \end{aligned}$$

The unreliability $U(T)$ represents the failure probability of the system modeled by the fault tree and is crucial to providing safety and availability guarantees. The expression in Definition 3 becomes too large to handle for large FTs very quickly; thus it is important to find efficient solutions to the following problem.

### Problem 5

Given a FT $T$, calculate $U(T)$.

Unfortunately, we show that this problem is NP-hard, by showing that the so-called *minimal cut set problem*, which is known to be NP-hard [11], can be reduced to it. The minimal cut set problem is stated as follows: Consider an unaugmented FT $T' = (V,E,\gamma )$, i.e., a FT without $\textrm{p}$ specified. This does not affect the definition of the structure function or cut sets. For $\vec {\sigma },\vec {\sigma }' \in {\mathbb {B}}^{\operatorname {BE}_{T'}}$, we write $\vec {\sigma } \sqsubseteq \vec {\sigma }'$ if $\sigma _v \le \sigma '_v$ for all $v \in \operatorname {BE}_{T'}$. A cut set $\vec {\sigma }$ is called *minimal* if there does not exist another cut set $\vec {\sigma }'$ with $\vec {\sigma }' \sqsubset \vec {\sigma }$.

### Example 6

In Example 4, we saw that the cut sets of the FT of Fig. 1 are 010,011,101,110,111. Of these 010 and 110 are minimal with respect to $\sqsubseteq$, so these are the minimal cut set.

The following problem is NP-hard [11]:

### Problem 7

Given an unaugmented FT $T'$, find a minimal cut set.

Therefore, the following result proves that Problem 5 is NP-hard:

### Theorem 8

Problem 7 can be reduced to Problem 5 in polynomial time.

### Proof

Let $$T' = (V,E,\gamma )$$ be an unaugmented FT, and take any enumeration $$\operatorname {BE}_{T'} = \{v_0,\ldots ,v_{n-1}\}$$. Define $$\textrm{p}\!:\operatorname {BE}_{T'} \rightarrow [0,1]$$ by $$\textrm{p}(v_i) = 10^{-2^i}$$, and let $$T = (V,E,$$$$\gamma ,\textrm{p})$$ be the resulting augmented fault tree. Let $$M \subseteq \operatorname {CS}_{T}$$ be the set of minimal cut sets. For $$\vec {\sigma } \in M$$, define $$\kappa (\vec {\sigma }) = -\log _{10}\left( \prod _{f_b = \texttt{1}} \textrm{p}(b)\right) $$; let $$\vec {\tau } \in M$$ be such that $$\kappa (\vec {\tau })$$ is minimal.

Let $\vec {\Sigma }$ be the random variable in ${\mathbb {B}}^{\operatorname {BE}_{T}}$ defined in Definition 3; then $\mathbb {P}(\vec {\sigma } \sqsubseteq \vec {\Sigma }) = 10^{-\kappa (\vec {\sigma })}$ for all $\vec {\sigma } \in M$. Since $\vec {\Sigma } \in \operatorname {CS}_{T}$ if and only if there is a $\vec {\sigma } \in M$ such that $\vec {\sigma } \sqsubseteq \vec {\Sigma }$, it follows that

1 $$\begin{aligned} 10^{-\kappa (\vec {\tau })} = \mathbb {P}(\vec {\tau } \sqsubseteq \vec {\Sigma }) \le \mathbb {P}(\exists \vec {\sigma } \in M:\vec {\sigma } \sqsubseteq \vec {\Sigma }) = U(T) . \end{aligned}$$

Furthermore, we also get

2 $$\begin{aligned} U(T)&= \mathbb {P}(\exists \vec {\sigma } \in M:\vec {\sigma } \sqsubseteq \vec {\Sigma }) \nonumber \\ &\le \sum _{\vec {\sigma } \in M} \mathbb {P}(\vec {\sigma } \sqsubseteq \vec {\Sigma }) = \sum _{\vec {\sigma } \in M} 10^{-\kappa (\vec {\sigma })}. \end{aligned}$$

We now give an upper bound on $\sum _{\vec {\sigma } \in M} 10^{-\kappa (\vec {\sigma })}$ in terms of $\kappa (\vec {\tau })$. If $\{v \in \operatorname {BE}_{T} \mid \sigma _v = \mathrm{1}\} = \{v_{i_1},\ldots ,v_{i_k}\}$, then $\kappa (\vec {\sigma }) = \sum _{j=1}^k 2^{i_j}$. Thus, each $\kappa (\vec {\sigma })$ is an integer, whose binary representation is equal to $\vec {\sigma }$ (i.e., its digit for $2^i$ indicates whether $\sigma _{v_i} = \texttt{1}$). As a result, the set $\{\kappa (\vec {\sigma }) \mid \vec {\sigma } \in M\}$ is a set of distinct integers, the least of which is $\kappa (\vec {\tau })$. Hence

3 $$\begin{aligned} \sum _{\vec {\sigma } \in M} 10^{-\kappa (\vec {\sigma })} \le \sum _{i=0}^{|M|-1} 10^{-\kappa (\vec {\tau })-i} < 10^{-\kappa (\vec {\tau })+1} \end{aligned}$$

From (1), (2), (3) it follows that $-\lfloor \log _{10}(U(T)) \rfloor = \kappa (\vec {\tau })$. Thus from $U(T)$ we find $\kappa (\vec {\tau })$ in polynomial time, and since $\vec {\tau }$ is the binary representation of the integer $\kappa (\vec {\tau })$, we also find the MCS $\vec {\tau }$. $\square$

### Existing $U(T)$ Algorithms

Two prominent algorithms exist for calculating *U*(*T*). The first one calculates, for each node $v$, the probability $g_v = \mathbb {P}(\operatorname {S}_{T}(v,\vec {\Sigma }) = \texttt{1})$ bottom-up. For BEs one has $g_v = \textrm{p}(v)$. For an AND-gate, one has $g_v = \prod _{w \in \operatorname {ch}(v)} g_w$ as long as the events $\operatorname {S}_{T}(w,\vec {\Sigma }) = \texttt{1}$ are independent as $w$ ranges over all children of $v$. This happens when no two children of *v* have any shared descendants. For OR-gates one has $g_v = 1-\prod _{w \in \operatorname {ch}(v)} (1-g_w)$ in the same manner. This induces a linear-time algorithm that calculates $g_v$ bottom-up. Unfortunately, this algorithm only works for FTs that have a tree structure: If there exists a node with multiple parents, the independence assumption will be violated at some point in the calculation.

The second algorithm [11] works for general FTs, by translating the Boolean function $\operatorname {S}_{T}(\operatorname {R}_{T},-)$ into a *binary decision diagram*. This is a directed acyclic graph in which the evaluation of the function at a boolean vector is represented by a path through the graph. After the BDD is found, $U(T)$ can be calculated using a bottom-up algorithm on the BDD, whose time complexity is linear in the size of the BDD. Unfortunately, the size of the BDD is worst-case exponential, although this worst case is seldomly attained in practice [13, 37]. The BDD depends on the choice of a linear ordering of the variables; finding the order that minimizes BDD size is an NP-hard problem, although heuristics exist [38, 39].

## An Example of Our Method

**Fig. 2:**
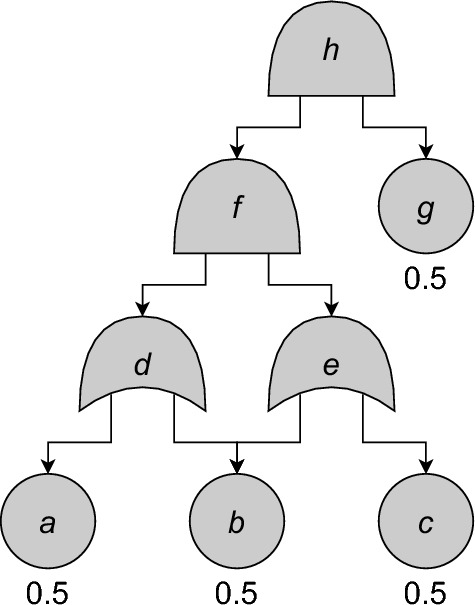
An example FT with failure probabilities. From [3, Fig. 2]. See Fig. 1 for gate types

Before we dive into the details of our method, we go through an example to showcase how the method works and to motivate the technical sections.

Consider the FT of Fig. 2. Going bottom-up, we calculate the failure probability $g_v$ of each node $v$: thus for the BEs we have $g_a = g_b = g_c = g_g = 0.5$. For *d*, the bottom-up method dictates that we should calculate $g_d = g_a+g_b-g_ag_b$. However, this will cause problems at $f$, since $d$ and $e$ share $b$ as child, and so their failure probabilities will not be independent. Thus, at $d$, we modify $g_d$ to ‘remember’ its dependence on $b$. We do so by introducing a formal variable $\mathsf{L}_{b}$ representing $b$’s failure probability, yielding $g_d = g_a+\mathsf{L}_{b}-g_a\mathsf{L}_{b} = 0.5+0.5\mathsf{L}_{b}$. Analogously we get $g_e = 0.5+0.5\mathsf{L}_{b}$. Note that we only introduce a formal variable for $b$, and not for $a$ and $c$, as these only have one parent node and thus will never appear twice in the same calculation.

At $$f$$, we calculate $$g_f = g_dg_e = 0.25+0.5\mathsf{L}_{b}+$$$$0.25\mathsf{L}_{b}^2$$. Here we introduce the rule $$\mathsf{L}_{b}^2 = \mathsf{L}_{b}$$, so that $$g_f = 0.25+0.75\mathsf{L}_{b}$$. The idea behind this is that since $$\mathsf{L}_{b}$$ represents $$\mathbb {P}(\operatorname {S}_{T}(b,\vec {\Sigma }) = \texttt{1})$$, the term $$\mathsf{L}_{b}^2$$ actually represents the probability $$\mathbb {P}(\operatorname {S}_{T}(b,\vec {\Sigma }) = \texttt{1}\wedge \operatorname {S}_{T}(b,\vec {\Sigma }) = \texttt{1})$$. This is equal to $$\mathbb {P}(\operatorname {S}_{T}(b,\vec {\Sigma }) = \texttt{1})$$, so $$\mathsf{L}_{b}^2=\mathsf{L}_{b}$$.

At this point, we see from the graph structure that *b* cannot appear twice in the same calculation any more. Thus we can safely substitute $g_b=0.5$ for $\mathsf{L}_{b}$ in $g_f = 0.25+0.75\mathsf{L}_{b}$, giving us $g_f = 0.625$. Finally, we get $g_h = g_fg_g = 0.3125$.

In the following sections, we introduce two mathematical tools needed to apply this method in greater generality. In “Preliminaries I: Dominators” we review the graph-theoretic notion of dominators, which will tell us for which nodes we need to introduce formal variables and at what point they can be substituted away. In “Preliminaries II: Squarefree Polynomial Algebras” we formalize the polynomial algebra in which our arithmetic takes place.

## Preliminaries I: Dominators

In this section we review the concept of dominators and apply them to FTs. This will be needed in our algorithm to determine at what point in the bottom-up calculation, outlined in the previous section, we can replace a formal variable $\mathsf{L}_{v}$ with the expression $g_v$. Informally, a dominator of $v$ is present on all paths from the root to $v$.

### Definition 9

[40] Let $T = (V,E,\gamma ,\textrm{p})$ be a FT.

1. Define a partial order $\sqsubseteq$ on $V$ by $x \preceq y$ iff there is a path $y \rightarrow x$ in *T*.
2. Given two nodes $v,w \in V$, we say that *w* *dominates* *v* if $v \prec w$ and every path $\operatorname {R}_{T} \rightarrow v$ in $T$ contains $w$.

The set of dominators of a node is nonempty and has a minimum:

### Lemma 10

[41] If $v \ne \operatorname {R}_{T}$, then there is a unique $w$ dominating $v$ such that each $w'$ dominating $v$ satisfies $w \preceq w'$; this $w$ is called the *immediate dominator of *$v$, denoted $w = \operatorname {id}(v)$. $\square$

### Example 11

In Fig. 2, the dominators of $a$ are $d$, $f$, and $h$. These satisfy $d \preceq f \preceq h$, so $\operatorname {id}(a) = d$. The dominators of $b$ are $f$ and $h$ with $f \preceq h$, so $\operatorname {id}(b) = f$. Note that $d$ is not a dominator of $b$, since the path $h \rightarrow f \rightarrow e \rightarrow b$ does not pass through *d*.

The immediate dominator interests us since, as we will see later, at $\operatorname {id}(v)$ we can replace $\mathsf{L}_{v}$ by $g_v$. The following result relates the relative position of $v$ and $w$ to that of their immediate dominators.

### Lemma 12

If $v \prec w$, then either $\operatorname {id}(v) \preceq w$ or $\operatorname {id}(w) \preceq \operatorname {id}(v)$.

### Proof

Suppose $\operatorname {id}(v) \not \preceq w$. Since $v \prec w$, any path $\operatorname {R}_{T} \rightarrow w$ can be extended to a path $\operatorname {R}_{T} \rightarrow v$; hence $\operatorname {id}(v)$ lies on the extended path. Since $\operatorname {id}(v) \not \preceq w$, it cannot lie on the subpath $w \rightarrow v$, so it lies on the path $\operatorname {R}_{T} \rightarrow w$. Since this is true for every path $\operatorname {R}_{T} \rightarrow w$, it follows that $\operatorname {id}(v)$ is a dominator of *w*; hence $\operatorname {id}(w) \preceq \operatorname {id}(v)$. $\square$

To use these definitions in an algorithmic context, we use the following result:

### Theorem 13

[41] Given $T$, there exists an algorithm of time complexity $\mathcal {O}(|E|)$ that finds $\operatorname {id}(v)$ for each $v \in V$. $\square$

## Preliminaries II: Squarefree Polynomial Algebras

In this second preliminary section, we formally define the algebras in which our calculations take place, and define their arithmetic. They are similar to multivariate polynomial algebras, except in every monomial every variable can have degree at most 1.

This section is quite dense in order to formally define all the necessary mathematics. In practice, these act like normal polynomials with respect to addition, multiplication, and substitution, except that one must consistently apply the rule of arithmetic that $$\mathsf{L}_x^2 = \mathsf{L}_x$$ for all $$$x$$$. If one is comfortable with that explanation, it is safe to skip to “Real-Valued Boolean Functions”, where the relation to boolean-input functions is discussed. However, understanding this section is necessary in order to understand the proof of this paper’s main result, Theorem 29.

### Definition 14

Let *X* be a finite set. We define the *squarefree real polynomial algebra over X* to be the algebra $\mathcal {A}(X)$ consisting of formal sums

$$ \alpha = \sum _{Y \subseteq X} \alpha _Y \prod _{x \in Y} \mathsf{L}_{x}, $$

where the $\mathsf{L}_{x}$ are formal variables and $\alpha _Y \in \mathbb {R}$.^1^ Addition and multiplication are as normal polynomials, except that they are subject to the law $\mathsf{L}_{x}^2 = \mathsf{L}_{x}$ for all $x \in X$; that is,

$$\begin{aligned} (\alpha +\beta )_Y&= \alpha _Y + \beta _Y,\\ (\alpha \cdot \beta )_Y&= \mathop{\sum}_{\substack{Y',Y'' \subseteq X:\\ Y'\cup Y'' = Y}} \alpha _{Y'}\beta _{Y''}. \end{aligned}$$

### Example 15

Let $X = \{x,y,z\}$. Let $\alpha = 2+\mathsf{L}_{x}+\mathsf{L}_{y}$ and $\beta = \mathsf{L}_{x}+3\mathsf{L}_{x}\mathsf{L}_{z}$. Coefficientwise, $\alpha$ is described as

$$\begin{aligned} \alpha _Y&= {\left\{ \begin{array}{ll} 2, & \text { if } Y = \varnothing ,\\ 1, & \text { if } Y = \{x\} \text { or } Y = \{y\},\\ 0, & \text { otherwise .} \end{array}\right. }\\ \beta _Y&= {\left\{ \begin{array}{ll} 3, & \text { if } Y = \{x,z\} ,\\ 1, & \text { if } Y = \{x\} ,\\ 0, & \text { otherwise .} \end{array}\right. } \end{aligned}$$

Furthermore, $$\alpha +\beta = 2+2\mathsf{L}_{x}+\mathsf{L}_{y}+3\mathsf{L}_{x}\mathsf{L}_{z}$$, and

$$\begin{aligned} \alpha \cdot \beta&= \alpha \cdot \mathsf{L}_{x} + \alpha \cdot (3\mathsf{L}_{x}\mathsf{L}_{z}) \\&= (2\mathsf{L}_{x}+\mathsf{L}_{x}^2+\mathsf{L}_{x}\mathsf{L}_{y}) + (6\mathsf{L}_{x}\mathsf{L}_{z}+3\mathsf{L}_{x}^2\mathsf{L}_{z}+3\mathsf{L}_{x}\mathsf{L}_{y}\mathsf{L}_{z}) \\&= (2\mathsf{L}_{x}+\mathsf{L}_{x}+\mathsf{L}_{x}\mathsf{L}_{y}) + (6\mathsf{L}_{x}\mathsf{L}_{z}+3\mathsf{L}_{x}\mathsf{L}_{z}+3\mathsf{L}_{x}\mathsf{L}_{y}\mathsf{L}_{z}) \\&= 3\mathsf{L}_{x}+\mathsf{L}_{x}\mathsf{L}_{y}+9\mathsf{L}_{x}\mathsf{L}_{z}+3\mathsf{L}_{x}\mathsf{L}_{y}\mathsf{L}_{z}. \end{aligned}$$

In particular, $(\alpha \cdot \beta )_{\{x\}} = 3$. We can also get this from the formal definition:

$$\begin{aligned} (\alpha \cdot \beta )_{\{x\}}&=\mathop{\sum}_{\substack{Y',Y'' \subseteq X:\\ Y'\cup Y'' = \{x\}}} \alpha _{Y'}\beta _{Y''}\\&= \alpha _{\varnothing }\beta _{\{x\}}+\alpha _{\{x\}}\beta _{\varnothing }+\alpha _{\{x\}}\beta _{\{x\}} \\&= 2\cdot 1 + 1 \cdot 0 + 1 \cdot 1 = 3. \end{aligned}$$

Furthermore, $\mathcal {A}(X)$ is a $2^3$-dimensional real vector space: one example of a basis is $\{1,\ \mathsf{L}_{x},\ \mathsf{L}_{y},\ \mathsf{L}_{z},\ \mathsf{L}_{x}\mathsf{L}_{y},\ \mathsf{L}_{x}\mathsf{L}_{z},\ \mathsf{L}_{y}\mathsf{L}_{z},\ \mathsf{L}_{x}\mathsf{L}_{y}\mathsf{L}_{z}\}$.

If $X \subseteq X'$, an $\alpha \in \mathcal {A}(X)$ can also be considered an element of $\mathcal {A}(X')$, by taking $\alpha _{Y} = 0$ whenever $Y \not \subseteq X$. For two sets *X* and *Y* this also allows us to add and multiply $\alpha \in \mathcal {A}(X)$ and $\beta \in \mathcal {A}(Y)$, by considering both to be elements of $\mathcal {A}(X \cup Y)$. In the rest of this paper we will do so without comment.

Besides multiplication and addition, the third important operation is the substitution of a formal variable by a polynomial. This works the same as with regular polynomials.

### Definition 16

Let *X*, $$$Y$$$ be finite sets and $$x \in X \setminus Y$$. Let $$\alpha \in \mathcal {A}(X)$$ and $$\beta \in \mathcal {A}(Y)$$. Then the *substitution* $$\alpha [\mathsf{L}_{x} \mapsto \beta ]$$ is the element of $$\mathcal {A}(X\setminus \{x\} \cup Y)$$ obtained by replacing all instances of $$\mathsf{L}_{x}$$ by $$\beta $$; more formally, $$\alpha [\mathsf{L}_{x} \mapsto \beta ]$$ is expressed as

$$\begin{aligned}&\beta \cdot \left( \mathop{\sum}_{\substack{ Z \subseteq X:\\ x \in Z }} \alpha _Z \prod _{x' \in Z\setminus \{x\}} \mathsf{L}_{x'}\right) + \mathop{\sum}_{\substack{ Z \subseteq X:\\ x \notin Z }} \alpha _Z \left( \prod _{x' \in Z} \mathsf{L}_{x'}\right) . \end{aligned}$$

In terms of coefficients this is expressed as

$$ \alpha [\mathsf{L}_{x} \mapsto \beta ]_Z = \alpha _Z + \mathop{\sum}_{\substack{ x \in Z' \subseteq X,\\ Z'' \subseteq Y:\\ Z' {\setminus } \{x\} \cup Z'' = Z}} \alpha _{Z'}\beta _{Z''} $$

where $$\alpha _Z = 0$$ if $$Z \not \subseteq X$$.

In this definition the multiplication and addition are of elements of $\mathcal {A}(X \setminus \{x\} \cup Y)$.

### Example 17

We continue Example 15, and wish to compute the substitution $\beta [\mathsf{L}_{z}\mapsto \alpha ]$; that is, we replace all instances of $\mathsf{L}_{z}$ in $\beta = \mathsf{L}_{x}+3\mathsf{L}_{x}\mathsf{L}_{z} \in \mathcal {A}(\{x,z\})$ with $\alpha = 2+\mathsf{L}_{x}+\mathsf{L}_{y} \in \mathcal {A}(\{x,y\})$. We get

$$\begin{aligned} \beta [\mathsf{L}_{z} \mapsto \alpha ]&= \mathsf{L}_{x}+3\mathsf{L}_{x} \cdot (2+\mathsf{L}_{x}+\mathsf{L}_{y}) \\&= 10\mathsf{L}_{x}+3\mathsf{L}_{x}\mathsf{L}_{y}. \end{aligned}$$

We can also get this from the coefficientwise expression of Definition 16: for instance, $\beta [\mathsf{L}_{z}\mapsto \alpha ]_{\{x\}}$ can be computed via

$$\begin{aligned} \beta [\mathsf{L}_{z}\mapsto \alpha ]_{\{x\}}&= \beta _{\{x\}} + \mathop{\sum}_{\substack{ z \in Z' \subseteq \{x,z\},\\ Z'' \subseteq \{x,y\}:\\ Z' \setminus \{z\} \cup Z'' = \{x\}}} \beta _{Z'}\alpha _{Z''}\\&= \beta _{\{x\}}+\beta _{\{z\}}\alpha _{\{x\}}+\beta _{\{x,z\}}\alpha _{\varnothing }+\beta _{\{z,x\}}\alpha _{\{x\}}\\&= 1 + 0\cdot 1 + 3\cdot 2 + 3 \cdot 1 = 10. \end{aligned}$$

Note that as an $\mathbb {R}$-algebra, one may identify $\mathcal {A}(X)$ with $K/I$, where $K = \mathbb {R}[\mathsf{L}_{x}:x \in X]$ is a free polynomial algebra and *I* is the ideal generated by the set $\{\mathsf{L}_{x}^2-\mathsf{L}_{x} \mid x \in X\}$. However, the substitution operation does not correspond to a ‘natural’ operation on on $K/I$.

### Rules of Arithmetic

In what follows, we will need three results on arithmetic in $\mathcal {A}(X)$.

#### An Alternative Expression for Substitution

The first result considerably simplifies the substitution operation.

##### Lemma 18

Let $X,Y,x,\alpha ,\beta$ be as in Definition 16. Then

$$ \alpha [\mathsf{L}_{x} \mapsto \beta ] = \alpha [\mathsf{L}_{x} \mapsto {1}]\cdot \beta + \alpha [\mathsf{L}_{x} \mapsto 0] \cdot ({1}-\beta ). $$

##### Proof

We have

$$\begin{aligned} \alpha [\mathsf{L}_{x} \mapsto \beta ]&= \mathop{\sum}_{\substack{ Y \subseteq X:\\ x \in Y}} \alpha _Y \left( \prod _{x' \in Y\setminus \{x\}} \mathsf{L}_{x'}\right) \\ &\quad \cdot \beta + \mathop{\sum}_{\substack{ Y \subseteq X:\\ x \notin Y}} \alpha _Y \left( \prod _{x' \in Y} \mathsf{L}_{x'}\right) \\&= \left( \mathop{\sum}_{\substack{Y \subseteq X:\\ x \in Y}} \alpha _Y \prod _{x' \in Y\setminus \{x\}} \mathsf{L}_{x'}+ \mathop{\sum}_{\substack{Y \subseteq X:\\ x \notin Y}} \alpha _Y \prod _{x' \in Y} \mathsf{L}_{x'}\right) \\ &\quad \cdot \beta + \left(\mathop{\sum}_{\substack{Y \subseteq X:\\ x \notin Y}} \alpha _Y \prod _{x' \in Y} \mathsf{L}_{x'}\right) \cdot (1-\beta )\\&= \left( \sum _{Y \subseteq X} \alpha _Y \prod _{x' \in Y\setminus \{x\}} \mathsf{L}_{x'}\right) \\ &\quad \cdot \beta + \left(\mathop{\sum}_{\substack{Y \subseteq X:\\ x \notin Y}} \alpha _Y \prod _{x' \in Y} \mathsf{L}_{x'}\right) \cdot (1-\beta )\\&= \alpha [\mathsf{L}_{x} \mapsto 1]\cdot \beta + \alpha [\mathsf{L}_{x} \mapsto 0] \cdot (1-\beta ). \end{aligned}$$

$$\square $$

##### Example 19

In Example 17, we saw that $\beta [\mathsf{L}_{z}\mapsto \alpha ] = 10\mathsf{L}_{x}+3\mathsf{L}_{x}\mathsf{L}_{y}$ when $\alpha = 2+\mathsf{L}_{x}+\mathsf{L}_{y}$ and $\beta = \mathsf{L}_{x}+3\mathsf{L}_{x}\mathsf{L}_{z}$. Indeed, Lemma 18 tells us that

$$\begin{aligned} \beta [\mathsf{L}_{z}\mapsto \alpha ]&= \beta [\mathsf{L}_{z} \mapsto 1]\cdot \alpha + \beta [\mathsf{L}_{z} \mapsto 0] \cdot (1-\alpha ) \\&= 4\mathsf{L}_{x}\cdot (2+\mathsf{L}_{x}+\mathsf{L}_{y})+\mathsf{L}_{x}\cdot (-1-\mathsf{L}_{x}-\mathsf{L}_{y})\\&= 8\mathsf{L}_{x}+4\mathsf{L}_{x}+4\mathsf{L}_{x}\mathsf{L}_{y}-\mathsf{L}_{x}-\mathsf{L}_{x}-\mathsf{L}_{x}\mathsf{L}_{y}\\&= 10\mathsf{L}_{x}+3\mathsf{L}_{x}\mathsf{L}_{y}. \end{aligned}$$

#### Arithmetic

The second result shows how substitution interacts with addition and multiplication.

##### Lemma 20

*Let* $\alpha _{1},\alpha _{2} \in \mathcal {A}(X)$, $\beta \in \mathcal {A}(Y)$, and $x \in X \setminus Y$. *Then:*

1. $$(\alpha _1+\alpha _2)[\mathsf{L}_{x} \mapsto \beta ] = \alpha _1[\mathsf{L}_{x} \mapsto \beta ] + \alpha _2[\mathsf{L}_{x} \mapsto \beta ]$$.
2. *If* $\beta ^2 = \beta$,* then furthermore* $(\alpha _1\alpha _2)[\mathsf{L}_{x} \mapsto \beta ] = \alpha _1[\mathsf{L}_{x} \mapsto \beta ] \cdot \alpha _2[\mathsf{L}_{x} \mapsto \beta ]$.

##### Example 21

Let $\alpha _1 = 2+\mathsf{L}_{x}, \alpha _2 = \mathsf{L}_{y}+3\mathsf{L}_{x}\mathsf{L}_{y}$, and $\beta = 1-\mathsf{L}_{y}$. Then

$$\begin{aligned} \alpha _1[\mathsf{L}_{x}\mapsto \beta ]&= 3-\mathsf{L}_{y},\\ \alpha _2[\mathsf{L}_{x}\mapsto \beta ]&= \mathsf{L}_{y}+3\mathsf{L}_{y}(1-\mathsf{L}_{y}) \\&= \mathsf{L}_{y}, \end{aligned}$$

so $\alpha _1[\mathsf{L}_{x}\mapsto \beta ]+\alpha _2[\mathsf{L}_{x}\mapsto \beta ] = 3$. On the other hand,

$$\begin{aligned} \alpha _1+\alpha _2&= 2+\mathsf{L}_{y}+(1+3\mathsf{L}_{y})\mathsf{L}_{x},\\ (\alpha _1+\alpha _2)[\mathsf{L}_{x} \mapsto \beta ]&= 2+\mathsf{L}_{y}+(1+3\mathsf{L}_{y})(1-\mathsf{L}_{y}) \\&= 2+\mathsf{L}_{y}+(1-\mathsf{L}_{y}) = 3. \end{aligned}$$

Furthermore, $\beta ^2 = 1-2\mathsf{L}_{y}+\mathsf{L}_{y}^2 = 1-\mathsf{L}_{y} = \beta$, so Lemma 20.2 applies. Indeed, we have

$$ \alpha _1[\mathsf{L}_{x}\mapsto \beta ] \cdot \alpha _2[\mathsf{L}_{x}\mapsto \beta ] = (3-\mathsf{L}_{y})\mathsf{L}_{y} = 2\mathsf{L}_{y}, $$

and

$$\begin{aligned} \alpha _1\alpha _2&= (2+\mathsf{L}_{x})(\mathsf{L}_{y}+3\mathsf{L}_{x}\mathsf{L}_{y})\\&= 2\mathsf{L}_{y}+10\mathsf{L}_{x}\mathsf{L}_{y},\\ (\alpha _1\alpha _2)[\mathsf{L}_{x}\mapsto \beta ]&= 2\mathsf{L}_{y}+10\mathsf{L}_{y}(1-\mathsf{L}_{y})\\&= 2\mathsf{L}_{y}. \end{aligned}$$

##### Example 22

Take $$\alpha _1 = \alpha _2 = \mathsf{L}_{x}$$, so $$\alpha _1\alpha _2 =$$$$ \mathsf{L}_{x}$$. Then for any $$\beta $$ we have $$(\alpha _1\alpha _2)[\mathsf{L}_{x} \mapsto \beta ] = \beta $$, and $$\alpha _1[\mathsf{L}_{x}\mapsto \beta ]\cdot \alpha _2[\mathsf{L}_{x} \mapsto \beta ] = \beta ^2$$. This shows the need for the assumption $$\beta ^2 = \beta $$ in Lemma 20.2.

##### Proof of Lemma 20

The first statement follows immediately from the coefficientwise definitions of addition and substitution. For the second statement, we prove it first for the special cases $\beta =1$ and $\beta =0$. In the first case, for each $Y \subseteq X \setminus \{x\}$ one has

$$ \alpha [\mathsf{L}_{x} \mapsto 1]_Y = \alpha _Y + \alpha _{Y \cup \{x\}}. $$

It follows that

$$\begin{aligned} (\alpha _1\alpha _2)[\mathsf{L}_{x} \mapsto 1]_Y&= (\alpha _1\alpha _2)_Y + (\alpha _1\alpha _2)_{Y \cup \{x\}} \\&= \mathop{\sum}_{\substack{Y_1,Y_2 \subseteq Y:\\ Y_1 \cup Y_2 = Y}} \alpha _{1,Y_1}\alpha _{2,Y_2} \\ &\quad + \mathop{\sum}_{\substack{Y'_1,Y'_2 \subseteq Y\cup \{x\}:\\ Y'_1 \cup Y'_2 = Y\cup \{x\}}} \alpha _{1,Y'_1}\alpha _{2,Y'_2}. \end{aligned}$$

In the second summation we can distinguish the cases $x \in Y'_1 \setminus Y'_2$, $x \in Y'_2 \setminus Y'_1$ and $x \in Y'_1 \cap Y'_2$. Substituting $Y_i:= Y'_i \setminus \{x\}$ in the second summation, we get

$$\begin{aligned} (\alpha _1\alpha _2)[\mathsf{L}_{x} \mapsto 1]_Y&= \sum _{\begin{array}{c} Y_1,Y_2 \subseteq Y:\\ Y_1 \cup Y_2 = Y \end{array}} \alpha _{1,Y_1}\alpha _{2,Y_2} \\&\quad + \sum _{\begin{array}{c} Y_1,Y_2 \subseteq Y:\\ Y_1 \cup Y_2 = Y \end{array}} \Big ( \alpha _{1,Y_1\cup \{x\}}\alpha _{2,Y_2} \\ &\quad +\alpha _{1,Y_1}\alpha _{2,Y_2\cup \{x\}}+\alpha _{1,Y_1\cup \{x\}}\alpha _{2,Y_2\cup \{x\}}\Big )\\&= \sum _{\begin{array}{c} Y_1,Y_2 \subseteq Y:\\ Y_1 \cup Y_2 = Y \end{array}} \Big ( \alpha _{1,Y_1}\alpha _{2,Y_2} + \alpha _{1,Y_1\cup \{x\}}\alpha _{2,Y_2} \\ &\quad +\alpha _{1,Y_1}\alpha _{2,Y_2\cup \{x\}}+\alpha _{1,Y_1\cup \{x\}}\alpha _{2,Y_2\cup \{x\}}\Big )\\&= \sum _{\begin{array}{c} Y_1,Y_2 \subseteq Y:\\ Y_1 \cup Y_2 = Y \end{array}} \alpha _1[\mathsf{L}_{x} \mapsto 1]_{Y_1}\alpha _2[\mathsf{L}_{x} \mapsto 1]_{Y_2}\\&= (\alpha _1[\mathsf{L}_{x} \mapsto 1]\cdot \alpha _2[\mathsf{L}_{x} \mapsto 1])_Y. \end{aligned}$$

This proves the statement for $\beta = 1$. The proof for $\beta = 0$ is analogous, this time using $\alpha [\mathsf{L}_{x} \mapsto 0]_Y = \alpha _Y$ for $Y \subseteq X \setminus \{x\}$.

Now we go to general $\beta$, for which we have

4 $$\begin{aligned}&\alpha _1[\mathsf{L}_{x} \mapsto \beta ] \cdot \alpha _2[\mathsf{L}_{x} \mapsto \beta ]\nonumber \\&\quad = \Big (\alpha _1[\mathsf{L}_{x} \mapsto 1]\cdot \beta + \alpha _1[\mathsf{L}_{x} \mapsto 0] \cdot (1-\beta )\Big ) \nonumber \\ &\qquad \cdot \Big (\alpha _2[\mathsf{L}_{x} \mapsto 1]\cdot \beta + \alpha _2[\mathsf{L}_{x} \mapsto 0] \cdot (1-\beta )\Big ) \end{aligned}$$

5 $$\begin{aligned}&\quad = \alpha _1[\mathsf{L}_{x} \mapsto 1]\cdot \alpha _2[\mathsf{L}_{x} \mapsto 1] \cdot \beta ^2 + \alpha _1[\mathsf{L}_{x} \mapsto 0] \nonumber \\ &\qquad \cdot \alpha _2[\mathsf{L}_{x} \mapsto 0] \cdot (1-2\beta +\beta ^2) \nonumber \\&\quad \quad + \Big (\alpha _1[\mathsf{L}_{x} \mapsto 1]\cdot \alpha _2[\mathsf{L}_{x} \mapsto 0] +\alpha _1[\mathsf{L}_{x} \mapsto 0] \nonumber \\ &\qquad \cdot \alpha _2[\mathsf{L}_{x} \mapsto 1]\Big ) \cdot (\beta -\beta ^2) \nonumber \\&\quad = \alpha _1[\mathsf{L}_{x} \mapsto 1]\cdot \alpha _2[\mathsf{L}_{x} \mapsto 1] \cdot \beta + \alpha _1[\mathsf{L}_{x} \mapsto 0] \nonumber \\ &\qquad \cdot \alpha _2[\mathsf{L}_{x} \mapsto 0] \cdot (1-\beta ) \end{aligned}$$

6 $$\begin{aligned}&\quad = (\alpha _1\alpha _2)[\mathsf{L}_{x} \mapsto 1] \cdot \beta + (\alpha _1\alpha _2)[\mathsf{L}_{x} \mapsto 0] \cdot (1-\beta ) \end{aligned}$$

7 $$\begin{aligned}&\quad = (\alpha _1\alpha _2)[\mathsf{L}_{x} \mapsto \beta ]. \end{aligned}$$

Here we used Lemma 18 in (4) and (7), the fact that $\beta ^2 = \beta$ in (5), and our result for $\beta = 0$ and $\beta = 1$ in (6). $\square$

#### Multiple Substitutions

The third result states that two substitution operations can be interchanged, as long as one does not substitute a variable present in the other:

##### Lemma 23

Let $$\alpha \in \mathcal {A}(X),\,\beta _{1} \in \mathcal {A}(Y_{1}),\,\beta _{2} \in \mathcal {A}(Y_{2}),\,$$ $$x_{1},x_{2} \in X \setminus (Y_{1} \cup Y_{2})$$. Then $$\alpha [\mathsf{L}_{x_{1}}\mapsto \beta _{1}][\mathsf{L}_{x_{2}} \mapsto \beta _{2}] $$$$ = \alpha [\mathsf{L}_{x_{2}} \mapsto \beta _{2}][\mathsf{L}_{x_{1}} \mapsto \beta _{1}]$$.

##### Example 24

Consider $$\alpha = \mathsf{L}_{x}+\mathsf{L}_{x}\mathsf{L}_{y}$$, $$\beta _1 = 3-\mathsf{L}_{z}$$, $$\beta _2 = \mathsf{L}_{z}+\mathsf{L}_{w}$$. then

$$\begin{aligned} \alpha [\mathsf{L}_{x}\mapsto \beta _1]&= (3-\mathsf{L}_{z})(1+\mathsf{L}_{y}),\\ \alpha [\mathsf{L}_{x}\mapsto \beta _1][\mathsf{L}_{y} \mapsto \beta _2]&= (3-\mathsf{L}_{z})(1+\mathsf{L}_{z}+\mathsf{L}_{w}),\\ \alpha [\mathsf{L}_{y} \mapsto \beta _2]&= \mathsf{L}_{x}(1+\mathsf{L}_{z}+\mathsf{L}_{w}),\\ \alpha [\mathsf{L}_{y} \mapsto \beta _2][\mathsf{L}_{x} \mapsto \beta _1]&= (3-\mathsf{L}_{z})(1+\mathsf{L}_{z}+\mathsf{L}_{w}). \end{aligned}$$

##### Example 25

We also give an example that shows that the requirement that the substituted variables do not appear in $\beta _1$ and $\beta _2$ is really necessary. We take the same $\alpha ,\beta _1$ as in Example 24, but we take $\beta _2 = \mathsf{L}_{x}+\mathsf{L}_{z}$. Then

$$\begin{aligned} \alpha [\mathsf{L}_{x}\mapsto \beta _1]&= (3-\mathsf{L}_{z})(1+\mathsf{L}_{y}),\\ \alpha [\mathsf{L}_{x}\mapsto \beta _1][\mathsf{L}_{y} \mapsto \beta _2]&= (3-\mathsf{L}_{z})(1+\mathsf{L}_{z}+\mathsf{L}_{x})\\&= 3+3\mathsf{L}_{x}+\mathsf{L}_{z}-\mathsf{L}_{x}\mathsf{L}_{z},\\ \alpha [\mathsf{L}_{y} \mapsto \beta _2]&= \mathsf{L}_{x}(1+\mathsf{L}_{z}+\mathsf{L}_{x}),\\ \alpha [\mathsf{L}_{y} \mapsto \beta _2][\mathsf{L}_{x} \mapsto \beta _1]&= (3-\mathsf{L}_{z})(1+\mathsf{L}_{z}+3-\mathsf{L}_{z})\\&= 12-4\mathsf{L}_{z}. \end{aligned}$$

Hence $$\alpha [\mathsf{L}_{x}\mapsto \beta _1][\mathsf{L}_{y} \mapsto \beta _2] \ne \alpha [\mathsf{L}_{y} \mapsto \beta _2][\mathsf{L}_{x} \mapsto \beta _1]$$.

##### Proof of Lemma 23

As for Lemma 20 we first prove this for $\beta _1,\beta _2 \in \{0,1\}$. First suppose $\beta _1 = \beta _2 = 1$. Using the rule $\alpha [\mathsf{L}_{x} \mapsto 1]_Y = \alpha _Y + \alpha _{Y \cup \{x\}}$ from the proof of Lemma 20, we find, for $Y \subseteq X \setminus \{x_1,x_2\}$:

$$\begin{aligned} \alpha [\mathsf{L}_{x_1} \mapsto 1][\mathsf{L}_{x_2} \mapsto 1]_Y&= \alpha [\mathsf{L}_{x_1} \mapsto 1]_Y + \alpha [\mathsf{L}_{x_1} \mapsto 1]_{Y \cup \{x_2\}} \\&= \alpha _Y + \alpha _{Y \cup \{x_1\}} + \alpha _{Y \cup \{x_2\}} \\ &\quad + \alpha _{Y \cup \{x_1,x_2\}}\\&= \alpha [\mathsf{L}_{x_2} \mapsto 1][\mathsf{L}_{x_1} \mapsto 1]_Y. \end{aligned}$$

The other cases for $\beta _1,\beta _2 \in \{0,1\}$ are completely analogous, also using the rule $\alpha [\mathsf{L}_{x} \mapsto 0]_Y = \alpha _Y$. Since the substitution order does not matter, we write e.g. $\alpha [\mathsf{L}_{x_1} \mapsto 1, \mathsf{L}_{x_2} \mapsto 0]$.

Now consider general $\beta _1,\beta _2$. Through repeated use of Lemmas 18 and 20 we find

$$\begin{aligned}&\alpha [\mathsf{L}_{x_1} \mapsto \beta _1][\mathsf{L}_{x_2} \mapsto \beta _2]\\&\quad = \alpha [\mathsf{L}_{x_1} \mapsto \beta _1][\mathsf{L}_{x_2} \mapsto 1]\cdot \beta _2 \\ &\qquad + \alpha [\mathsf{L}_{x_1} \mapsto \beta _1][\mathsf{L}_{x_2} \mapsto 0] \cdot (1-\beta _2) \\&\quad = \Big (\alpha [\mathsf{L}_{x_1} \mapsto 1] \cdot \beta _1 \\ &\qquad + \alpha [\mathsf{L}_{x_1} \mapsto 0] \cdot (1-\beta _1)\Big )[\mathsf{L}_{x_2} \mapsto 1]\cdot \beta _2\\&\qquad + \Big (\alpha [\mathsf{L}_{x_1} \mapsto 1] \cdot \beta _1 + \alpha [\mathsf{L}_{x_1} \mapsto 0] \cdot (1-\beta _1)\Big ) \\ &\qquad [\mathsf{L}_{x_2} \mapsto 0]\cdot (1-\beta _2)\\&\quad = \Big (\left( \alpha [\mathsf{L}_{x_1} \mapsto 1] \cdot \beta _1\right) [\mathsf{L}_{x_2} \mapsto 1] \\ &\qquad +\left( \alpha [\mathsf{L}_{x_1} \mapsto 0] \cdot (1-\beta _1)\right) [\mathsf{L}_{x_2} \mapsto 1] \Big )\cdot \beta _2\\&\qquad + \Big (\left( \alpha [\mathsf{L}_{x_1} \mapsto 1] \cdot \beta _1\right) [\mathsf{L}_{x_2} \mapsto 0]\\ &\qquad +\bigg (\alpha [\mathsf{L}_{x_1} \mapsto 0] \cdot (1-\beta _1)\bigg )[\mathsf{L}_{x_2} \mapsto 0] \Big )\cdot (1-\beta _2)\\&\quad = \Big (\alpha [\mathsf{L}_{x_1} \mapsto 1][\mathsf{L}_{x_2} \mapsto 1] \cdot \beta _1[\mathsf{L}_{x_2} \mapsto 1] \\ &\qquad +\alpha [\mathsf{L}_{x_1} \mapsto 0][\mathsf{L}_{x_2} \mapsto 1] \cdot (1-\beta _1)[\mathsf{L}_{x_2} \mapsto 1] \Big ) \\ &\qquad \cdot \beta _2 + \Big (\alpha [\mathsf{L}_{x_1} \mapsto 1][\mathsf{L}_{x_2} \mapsto 0] \cdot \beta _1[\mathsf{L}_{x_2} \mapsto 0] \\ &\qquad + \alpha [\mathsf{L}_{x_1} \mapsto 0][\mathsf{L}_{x_2} \mapsto 0] \cdot (1-\beta _1)[\mathsf{L}_{x_2} \mapsto 0] \Big )\cdot (1-\beta _2) \end{aligned}$$

By assumption, $x_2$ does not appear in $\beta _1$, so

$$\begin{aligned} \beta _1[\mathsf{L}_{x_2} \mapsto 1] = \beta _1[\mathsf{L}_{x_2} \mapsto 0] = \beta _1. \end{aligned}$$

So this last expression is equal to

$$\begin{aligned}&\Big (\alpha [\mathsf{L}_{x_1} \mapsto 1,\mathsf{L}_{x_2} \mapsto 1] \cdot \beta _1 \\ &\qquad+\alpha [\mathsf{L}_{x_1} \mapsto 0,\mathsf{L}_{x_2} \mapsto 1] \cdot (1-\beta _1) \Big )\cdot \beta _2\\&\qquad + \Big (\alpha [\mathsf{L}_{x_1} \mapsto 1,\mathsf{L}_{x_2} \mapsto 0] \cdot \beta _1 \quad \\ &\qquad+ \alpha [\mathsf{L}_{x_1} \mapsto 0,\mathsf{L}_{x_2} \mapsto 0] \cdot (1-\beta _1) \Big )\cdot (1-\beta _2)\\&\quad = \alpha [\mathsf{L}_{x_1} \mapsto 1,\mathsf{L}_{x_2} \mapsto 1] \cdot \beta _1\beta _2\\ &\qquad +\alpha [\mathsf{L}_{x_1} \mapsto 0,\mathsf{L}_{x_2} \mapsto 1] \cdot (1-\beta _1)\beta _2\\&\qquad + \alpha [\mathsf{L}_{x_1} \mapsto 1,\mathsf{L}_{x_2} \mapsto 0] \cdot \beta _1(1-\beta _2) \\ &\qquad + \alpha [\mathsf{L}_{x_1} \mapsto 0,\mathsf{L}_{x_2} \mapsto 0] \cdot (1-\beta _1)(1-\beta _2). \end{aligned}$$

Expanding $\alpha [\mathsf{L}_{x_2} \mapsto \beta _2][\mathsf{L}_{x_1} \mapsto \beta _1]$ in the same way yields the exact same result, so we conclude that $\alpha [\mathsf{L}_{x_1} \mapsto \beta _1][\mathsf{L}_{x_2} \mapsto \beta _2] = \alpha [\mathsf{L}_{x_2} \mapsto \beta _2][\mathsf{L}_{x_1} \mapsto \beta _1]$. $\square$

When this lemma applies and the order of substitutions does not matter, we will write expressions like $\alpha [\mathsf{L}_{x_1} \mapsto \beta _1,\mathsf{L}_{x_2} \mapsto \beta _2]$, or even $\alpha [\forall i \le n:\mathsf{L}_{x_i} \mapsto \beta _i]$.

### Real-Valued Boolean Functions

Just as regular polynomials represent multivariate real functions, we will use the elements of $\mathcal {A}(X)$ is to represent functions ${\mathbb {B}}^X \rightarrow \mathbb {R}$. The following result states that this can be done in a unique way. Since both elements of $\mathcal {A}(X)$ and functions ${\mathbb {B}}^X \rightarrow \mathbb {R}$ can be represented by $2^{|X|}$ real numbers, this should come as no surprise.

#### Theorem 26

*Let* *X* *be a finite set, and let* $g:{\mathbb {B}}^X \rightarrow \mathbb {R}$ *be any function. Then there exists a unique* $\langle g\rangle \in \mathcal {A}(X)$ *such that* $g(\vec {\tau } ) = \langle g\rangle [\forall x \in X:\mathsf{L}_{x} \mapsto \tau _x]$ *for all* $\vec {\tau } \in {\mathbb {B}}^X$.

#### Example 27

Let $X = \{x,y\}$. We represent elements $\vec {\tau } = {\mathbb {B}}^X$ as $\tau _x\tau _y$, so ${\mathbb {B}}^X = \{\mathrm{0}\mathrm{0},\mathrm{0}\mathrm{1},\mathrm{1}\mathrm{0},\mathrm{1}\mathrm{1}\}$. Consider the function $g:{\mathbb {B}}^X \rightarrow \mathbb {R}$ given by

$$ g(\mathrm{0}\mathrm{0}) = 2, \quad \quad g(\mathrm{0}\mathrm{1}) = 1, \quad \quad g(\mathrm{1}\mathrm{0}) = 3, \quad \quad g(\mathrm{1}\mathrm{1}) = 6. $$

We now wish to find $\langle g\rangle \in \mathcal {A}(X)$ such that $\langle g\rangle [x \mapsto \tau _x, y \mapsto \tau _y] = g(\tau _x\tau _y)$ for all $\vec {\tau } \in {\mathbb {B}}^X$. If $\langle g\rangle = k+l\mathsf{L}_{x}+m\mathsf{L}_{y}+n\mathsf{L}_{x}\mathsf{L}_{y}$, then this amounts to solving the system of linear equations

$$\begin{aligned} k&= 2,\\ k+m&= 1,\\ k+l&= 3,\\ k+l+m+n&= 6. \end{aligned}$$

A solution is $(k,l,m,n) = (2,1,-1,4)$, so $\langle g\rangle = 2+\mathsf{L}_{x}-\mathsf{L}_{y}+4\mathsf{L}_{x}\mathsf{L}_{y}$. In fact, this solution is unique, so $\langle g\rangle$ is unique.

#### Proof of Theorem 26

Let $\operatorname {Map}({\mathbb {B}}^{X},\mathbb {R})$ be the set of functions ${\mathbb {B}}^X \rightarrow \mathbb {R}$. Consider the map $\varrho \colon\mathcal {A}(X) \rightarrow \operatorname {Map}({\mathbb {B}}^{X},\mathbb {R})$ given by $\varrho (g)(\vec {\tau }) = g[\forall x \in X:\mathsf{L}_{x} \mapsto \tau _x]$ for all $g \in \mathcal {A}(X)$ and $\vec {\tau } \in {\mathbb {B}}^X$. We will show that this map is bijective; this completes the proof of the theorem, as its inverse is then the sought map $f \mapsto \langle f\rangle$.

Let $\sqsubseteq$ be the partial order on ${\mathbb {B}}^X$ given by $\vec {\tau } \sqsubseteq \vec {\tau }'$ if and only if $\tau _x \le \tau '_x$ for all $x \in X$. Furthermore, let ${\mathbb {B}}^X = \{\vec {\tau }^1,\ldots ,\vec {\tau }^{M}\}$ be an enumeration of ${\mathbb {B}}^X$ (so $M = 2^{|X|}$) such that $\vec {\tau }^i \sqsubseteq \vec {\tau }^j$ implies $i \le j$ for all $i,j \le M$. Then $\operatorname {Map}({\mathbb {B}}^X,\mathbb {R})$ can be identified with $\mathbb {R}^M$, sending *f* to the vector $(f(\tau _1),\ldots ,f(\tau _M))^{\intercal } \in \mathbb {R}^M$. We can also identify $\mathcal {A}(X)$ with $\mathbb {R}^M$, by identifying *g* with $(g_{X_1},\ldots ,g_{X_M})$; here $X^i = \{x \in X \mid \tau ^i_X = \texttt{1}\}$. By construction, one has $X^i \subseteq X^j$ if and only if $\tau ^i \sqsubseteq \tau ^j$.

Under these identifications, we can regard $\varrho$ as a map $\mathbb {R}^M \rightarrow \mathbb {R}^M$; we now investigate what form this map takes. Note that

$$\begin{aligned} \varrho (g)(\vec {\tau }^i)&= \sum _{X' \subseteq X} g_{X'}\left( \prod _{x \in X'} \tau ^{i}_{x}\right) . \end{aligned}$$

The latter product equals 1 if $X' \subseteq X_i$, and 0 otherwise; hence

8 $$\begin{aligned} \varrho (g)(\vec {\tau }^i)&= \sum _{j:X_{j} \subseteq X_{i}} g_{{X_j}}. \end{aligned}$$

Thus $\varrho$ is a linear map $\mathbb {R}^M \rightarrow \mathbb {R}^M$ and can be represented by a matrix *E*. By (8), one has

$$ E_{i,j} = {\left\{ \begin{array}{ll} 1, \text { if } \tau _i \sqsubseteq \tau _j,\\ 0, \text { otherwise.} \end{array}\right. } $$

In particular, *E* is lower triangular and the diagonal entries are all 1. It follows that *E* is invertible, hence $\varrho$ is bijective. $\square$

**Algorithm 1:**
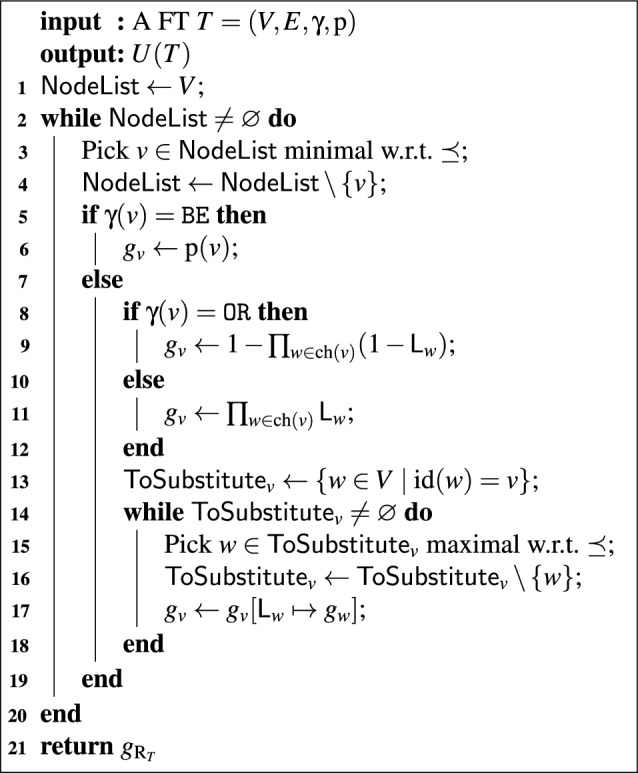
The algorithm $\mathtt{SFPA}(T)$. For an explanation, see “The Algorithm”.

## The Algorithm

We can now state our algorithm for calculating unreliability using the notation of the previous two sections. It is presented in Algorithm 1. The algorithm works bottom-up, assigning to each node $$$v$$$ a formal expression $$g_v \in \mathcal {A}(\{w \in V \mid w \prec v\})$$ representing the failure probability of $$$v$$$. In such a $$g_v$$, the formal variables $$\mathsf{L}_{w}$$ represent nodes with multiple paths from the root (), which we will also encounter further in the calculation.

The algorithm works as follows: working bottom-up (lines 1–4), the algorithm first assigns a $$g_v$$ of the most basic form to $$$v$$$ (lines 5–12): for a BE this is simply its failure probability $$\textrm{p}(v)$$, while for OR/AND-gates it is the expression for the failure probability in terms of the formal variables $$\mathsf{L}_{w}$$, where $$$v$$$ ranges over $$\operatorname {ch}(v)$$. After finding this expression of $$g_v$$, the algorithm then substitutes away all formal variables that do not come up later in the computation (lines 13–18). These are precisely the $$\mathsf{L}_{w}$$ for which $$\operatorname {id}(w) = v$$, as for these $$$w$$$ this is the point where we will not encounter other copies of $$\mathsf{L}_{w}$$ anymore. We replace each $$\mathsf{L}_{w}$$ with its associated expression $$g_w$$; we start with the $$$w$$$ closest to $$$v$$$, as these $$g_w$$ may contain other $$\mathsf{L}_{w'}$$ that also need to be substituted away. Finally, we return $$g_{\operatorname {R}_{T}}$$ (line 21). Now all formal variables have been substituted away, so $$g_{\operatorname {R}_{T}} \in \mathbb {R}$$. Note that this relies on finding $$\operatorname {id}(v)$$ for each $$$v$$$, which can be done in linear time by Theorem 13.

**Fig. 3:**
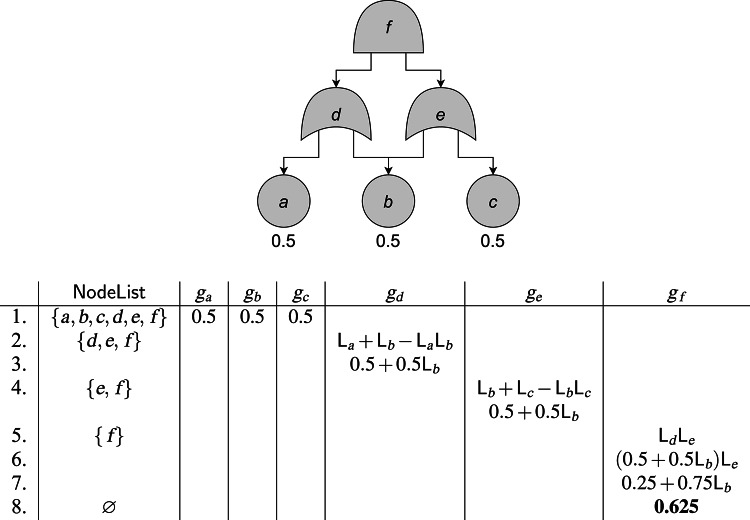
The application of $\mathsf{SFPA}$ on an example FT. For details, see Example 28. See Fig. 1 for gate types

### Example 28

We perform $\mathtt{SFPA}$ on the FT from Fig. 3:

1. We initialize $$\mathsf{NodeList} = \{a,b,c,d,e,f\}$$. Of these, $$$a$$$, $$$b$$$, $$$c$$$ are minimal with respect to $$\preceq $$. They are also all basic events, so on the first three iterations of the while-loop we set $$g_a = g_b = g_c = 0.5$$.
2. At this point $$\mathsf{NodeList} = \{d,e,f\}$$. Of these $$$d$$$ and $$$e$$$ are minimal; suppose we pick *d*. This is an OR-gate, so by line 9 we set $$g_d = 1-(1-\mathsf{L}_{a})(1-\mathsf{L}_{b}) = \mathsf{L}_{a}+\mathsf{L}_{b}-\mathsf{L}_{a}\mathsf{L}_{b}$$.
3. Next, in line 13, we have to find all vertices for which *d* is the immediate dominator. The only descendants of *d* are *a* and *b*, and $\operatorname {id}(a) = d$, $\operatorname {id}(b) = f$, so $\mathsf{ToSubstitute}_v = \{a\}$. In lines 14–19, we then set $g_d$ to $g_d[\mathsf{L}_{a}\mapsto g_a] = 0.5+0.5\mathsf{L}_{b}$.
4. Now $\mathsf{NodeList} = \{e,f\}$ with $e \preceq f$, so we pick *e*. Similarly to *d*, we get $g_e = 0.5+0.5\mathsf{L}_{b}$.
5. Now $\mathsf{NodeList} = \{f\}$, so we pick its unique element. We get $g_f = \mathsf{L}_{d}\mathsf{L}_{e}$ on line 11.
6. Next, $\mathsf{ToSubstitute}_f = \{b,d,e\}$, with $b \preceq d$ and $b \preceq e$. Thus in line 15, we first have to pick either *d* or *e* for the substitution step in line 17. Suppose we pick *d* first; we get $g_f = (0.5+0.5\mathsf{L}_{b})\mathsf{L}_{e}$.
7. Next, we have to pick *e*, and we get $g_f = (0.5+0.5\mathsf{L}_{b})^2 = 0.25+0.75\mathsf{L}_{b}$.
8. Finally, we substitute $g_b = 0.5$ for $\mathsf{L}_{b}$ and we get $g_f = 0.625$. Having finished our computation for $v = f$, the set $\mathsf{NodeList}$ is now empty, so we exit the while loop of lines 2–20, and we return $g_f = 0.625$, which is the unreliability of the FT.

The main theoretical result of this paper is the validity of Algorithm 1.

### Theorem 29

Let $T$ be a FT. Then $\mathtt{SFPA}(T) = U\!(\!T)$.        

We will prove this theorem in “Proof of Correctness”. First, we introduce a slight extension to the FT formalism that will be needed in the proof.

### Remark 30

The $g_v$ that are computed in Algorithm 1 represent polynomial expressions of the failure probabilities of intermediate gates; these can be converted to real numbers by appropriate substitution. In this way, we get additional information on weak points of the system. Furthermore, if we choose a basic event $b$ to systematically leave out of each $\mathsf{ToSubstitute}_v$, the outcome $g_{\operatorname {R}_{T}}$ is a linear polynomial expression in $\mathsf{L}_{b}$, which tells us how sensitive the overall failure probability is to the failure of *b*.

## Partially Controllable Fault Trees

In this section, we slightly extend the FT formalism for use in the proof of Theorem 29. The resulting objects, *partially controllable fault trees* (PCFTs), are just like regular FTs, except that certain BEs are labelled *controllable BEs*. Uncontrollable BEs do not have a fixed failure probability, but instead can be set to $\texttt{0}$ or $\texttt{1}$ at will. We emphasize that the concept of PCFTs does not correspond to an engineering reality, but is a mathematical construct needed for the proof of Theorem 29.

### Definition 31

An *partially controllable fault tree* (PCFT) is a tuple $T = (V,E,\gamma ,\textrm{p})$ where:

- ($V$, $E$) is a rooted directed acyclic graph;
- $\gamma$ is a function $\gamma \colon V \rightarrow \{\texttt{OR},\texttt{AND},\texttt{BE},\texttt{CBE}\}$ such that $\gamma (v) \in \{\texttt{BE},\texttt{CBE}\}$ iff *v* is a leaf;
- $\textrm{p}$ is a function $\textrm{p}\colon\operatorname {BE}_{T} \rightarrow [0,1]$, where $\operatorname {BE}_{T} = \{v \in V \mid \gamma (v) = \texttt{BE}\}$.

Similar to $\operatorname {BE}_{T}$ we define $\operatorname {CBE}_{T} = \{v \in V \mid \gamma (v) = \texttt{CBE}\}$. Since the failure of CBEs is not probabilistic, one can only speak of the failure probability of $T$ once one has set the states of the CBEs. Therefore, $U(T)$ is not a fixed probability, but a function ${\mathbb {B}}^{\operatorname {CBE}_{T}} \rightarrow [0,1]$.

### Definition 32

1. The structure function of $$$T$$$ is a map $$V \times {\mathbb {B}}^{\operatorname {BE}_{T}} \times {\mathbb {B}}^{\operatorname {CBE}_{T}} \rightarrow {\mathbb {B}}$$ defined by
  $$ \operatorname {S}_{T}(v,\vec {\sigma },\vec {\tau }) = {\left\{ \begin{array}{ll} \sigma _v, & \textrm{if}\:\gamma (v) = \texttt{BE},\\ \tau _v, & \textrm{if}\: \gamma (v) = \texttt{CBE}, \\ \bigvee _{w \in \operatorname {ch}(v)} \operatorname {S}_{T}(w,\vec {\sigma },\vec {\tau }), & \textrm{if}\: \gamma (v) = \texttt{OR},\\ \bigwedge _{w \in \operatorname {ch}(v)} \operatorname {S}_{T}(w,\vec {\sigma },\vec {\tau }), & \textrm{if}\: \gamma (v) = \texttt{AND}. \end{array}\right. } $$
2. Let $\vec {\Sigma }\in {\mathbb {B}}^{\operatorname {BE}_{T}}$ be a random variable so that $\Sigma _v$ is Bernoulli distributed with $\mathbb {P}(\Sigma _v = \texttt{1}) = \textrm{p}(v)$ for each $v \in \operatorname {BE}_{T}$, and all $\Sigma _v$ are independent. Then the *unreliability* of *T* is the function $U(T)\colon{\mathbb {B}}^{\operatorname {CBE}_{T}} \rightarrow [0,1]$ given by
  $$ U(T)(\vec {\tau }) = \mathbb {P}(\operatorname {S}_{T}(\operatorname {R}_{T},\vec {\Sigma },\vec {\tau }) = \texttt{1}). $$

In light of Theorem 26, the function $U(T)\colon{\mathbb {B}}^{\operatorname {CBE}_{T}} \rightarrow {\mathbb {B}}$ is described by its associated polynomial

$$ \langle U(T) \rangle \in \mathcal {A}(\operatorname {CBE}_{T}). $$

**Fig. 4:**
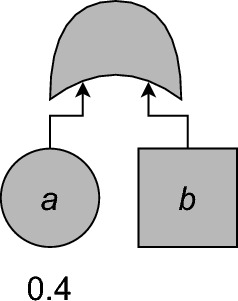
The PCFT of Example 33. *a* is a basic event (BE), while $b$ is a controllable basic event (CBE). From [3, Fig. 3]. See Fig. 1 for gate types

### Example 33

Consider the PCFT $\texttt{OR}(a,b)$, where $\gamma (a) = \texttt{BE}$ and $\gamma (b) = \texttt{CBE}$ (see Fig. 4) and $\textrm{p}(a) = 0.4$. Then ${\mathbb {B}}^{\operatorname {BE}_{T}} = {\mathbb {B}}^{\operatorname {CBE}_{T}} = {\mathbb {B}}$, and $\operatorname {S}_{T}(\operatorname {R}_{T},\sigma ,\tau ) = \sigma _a \vee \tau _b$. Since $\mathbb {P}(\Sigma _a = 1) = \textrm{p}(a) = 0.4$, it follows that

$$ U(T)(\tau ) = {\left\{ \begin{array}{ll} 0.4,& \text { if } \tau _b = \texttt{0},\\ 1,& \text { if } \tau _b = \texttt{1}. \end{array}\right. } $$

As a polynomial this is $\langle U(T) \rangle = 0.4+0.6\mathsf{L}_{b}$.

### Quasimodular Composition

Having expressed a PCFT $T$ as a polynomial $\langle U(T) \rangle$, we now relate substitution operations on such polynomials to graph-theoretic operations on PCFTs. The key concept on the PCFT side is *quasimodular composition*, which is defined as follows.

#### Definition 34

**Fig. 5:**
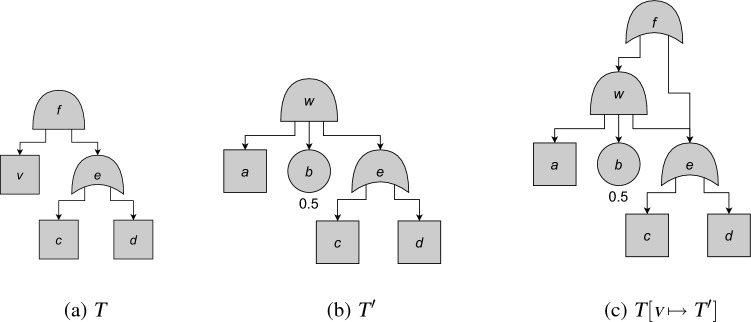
An example of quasimodular composition. Square nodes are CBEs. From [3, Fig. 4]

Let $T = (V,E,\gamma ,\textrm{p})$ and $T' = (V',E',\gamma ',\textrm{p}')$ where $V$ and $V'$ are not necessarily disjoint, such that $E,\gamma ,\textrm{p},\operatorname {ch}$ coincide with $E',\gamma ',\textrm{p}',\operatorname {ch}$ on $V \cap V'$. Let $v \in \operatorname {CBE}_{T} \setminus V'$, and assume that $\operatorname {BE}_{T} \cap \operatorname {BE}_{T'} = \varnothing$ (see Fig. 5). Then the *quasimodular composition* $T[v \mapsto T']$ of $T$ and $T'$ in $v$ is the PCFT obtained by replacing $v$ in *T* by the entire PCFT $T'$, rerouting all edges originally to *v* to $\operatorname {R}_{T'}$ instead. Formally, if $T[v \mapsto T'] = (V'',E'',\gamma '',\textrm{p}'')$, then

$$\begin{aligned} V''&= V \cup V' \setminus \{v\},\\ E''&= (E \setminus (V \times \{v\})) \cup E' \cup \{w\operatorname {R}_{T'} \mid wv \in E\},\\ \gamma ''(w)&= {\left\{ \begin{array}{ll} \gamma (w), & { if } w \in V ,\\ \gamma '(w), & { if } w \in V' \end{array}\right. }\\ \textrm{p}''(w)&= {\left\{ \begin{array}{ll} \textrm{p}(w), & { if } w \in V ,\\ \textrm{p}'(w), & { if } w \in V' \end{array}\right. }. \end{aligned}$$

#### Example 35

We discuss the example of Fig. 5 in more detail. Formally, the two PCFTs $T = (V,E,\gamma ,\textrm{p})$ and $T' = (V',E',\gamma ',\textrm{p}')$ are defined as follows ($\textrm{p}$ is empty):

$$\begin{aligned} V&= \{c, d,e,v,f\},\\ E&= \{ec, ed,fe,fv\}, \\ \gamma(e)=\gamma(f)&=\mathtt{OR},\\ \gamma(c)=\gamma(d)=\gamma(v)&=\mathtt{CBE}, \\V'&= \{a,b,c,d,e,w\},\\ E'&= \{ec, ed,wa,wb,we\}, \\\gamma'(v)&=\mathtt{AND},\\ \gamma'(e)=\gamma'(v)&=\mathtt{OR},\\ \gamma'(b)&=\mathtt{BE}, \\ \gamma'(a)=\gamma'(c)=\gamma'(d)&=\mathtt{CBE}, \\ \textrm{p}'(b)&=\textrm{0.5}.\end{aligned}$$

Then $V \cap V' = \{c,d,e\}$, and indeed $E$ and $E'$, $\gamma$ and $\gamma '$, and $\textrm{p}$ and $\textrm{p}'$ overlap on this. We then obtain $T[v \mapsto T']$ by replacing *v* in *T* by *w*, and also adding all of $w$’s descendants and their connection to $w$. This is reflected in $T[v \mapsto T'] = (V'',E'',\gamma '',\textrm{p}'')$ as

$$\begin{aligned} V''&= \{a, b,c, d,e,f,w\},\\ E''&= \{ec, ed,fe,wa, wb,we\}, \\\gamma''(v)&=\mathtt{AND},\\ \gamma''(e)=\gamma''(f)&=\mathtt{OR},\\ \gamma''(b)&=\mathtt{BE}, \\ \gamma''(a)=\gamma''(c)=\gamma''(d)&=\mathtt{CBE}, \\ \textrm{p}''(b)&=\textrm{0.5}.\end{aligned}$$

The concept of quasimodular composition of PCFTs is closely related to modular composition of FTs [13], in which a basic event of a FT is replaced by another FT. However, in modular composition $T$ and $T'$ may not share any nodes, while in quasimodular composition they may share CBEs, as well as any internal nodes. Due to the condition that these internal nodes must have the same children in $T$ and $T'$, any shared internal nodes may not have any BE descendants.

The following result states that substitution on the polynomial level precisely corresponds to quasimodular composition on the PCFT level; it is the key ingredient to the proof of Theorem 29.

#### Theorem 36

Let $T,T',v$ be as in Definition 34 and let $T'' = T[v \mapsto T']$ be their quasimodular composition. Then $\operatorname {CBE}_{T''} = \operatorname {CBE}_{T}\setminus \{v\} \cup \operatorname {CBE}_{T'}$, and as elements of $\mathcal {A}(\operatorname {CBE}_{T''})$ one has

$$ \langle U(T'')\rangle = \langle U(T)\rangle [\mathsf{L}_{v} \mapsto \langle U(T')\rangle ]. $$

#### Example 37

We continue Example 35. We can regard $U(T)$as a function ${\mathbb {B}}^{\{c,d,v\}} \rightarrow [0,1]$ given by $U(T)(\tau _c,\tau _d,\tau _v) = \tau _c \vee \tau _d \vee \tau _v$. This is equivalently expressed by the squarefree polynomial

$$\begin{aligned} \langle U(T)\rangle&= 1-(1-\mathsf{L}_{c})(1-\mathsf{L}_{d})(1-\mathsf{L}_{v}) \\ &= \mathsf{L}_{c}+\mathsf{L}_{d}+\mathsf{L}_{v}-\mathsf{L}_{c}\mathsf{L}_{d}-\mathsf{L}_{c}\mathsf{L}_{v}-\mathsf{L}_{d}\mathsf{L}_{v}+\mathsf{L}_{c}\mathsf{L}_{d}\mathsf{L}_{v}. \end{aligned}$$

The expression for $U(T'):{\mathbb {B}}^{\{a,c,d\}} \rightarrow [0,1]$ is a bit more complicated: the root is activated if $c$ or $d$ is activated, $a$ is activated, and *b* is activated (probability 0.5); this is expressed as $U(T')(\tau _a,\tau _c,\tau _d) = 0.5\cdot (\tau _a \wedge (\tau _c \vee \tau _d))$. In terms of squarefree polynomials, we get $\langle U(T')\rangle = 0.5\mathsf{L}_{a}(\mathsf{L}_{c}+\mathsf{L}_{d}-\mathsf{L}_{c}\mathsf{L}_{d})$. To get $\langle U(T[v \mapsto T'])\rangle$, we have to substitute $\mathsf{L}_{v}$ for this in the expression for $\langle U(T)\rangle$. From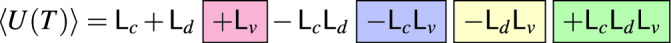 We get
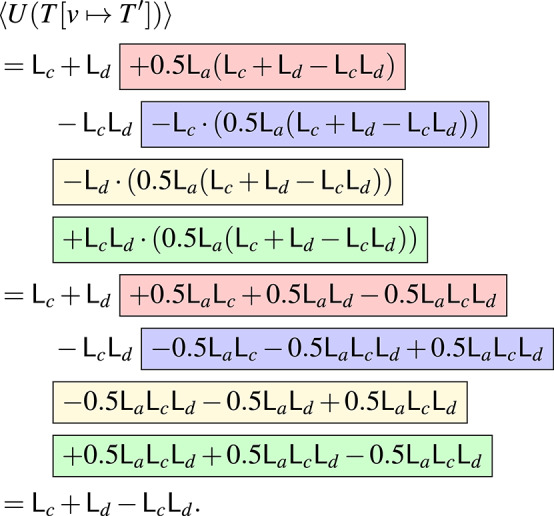


We can understand this rather simplified expression from Fig. 5: to activate $$v$$, at least $$e$$ needs to be activated. But, once $$e$$ is activated, $$v$$’s activation does not play a role for $$f $$’s activation any more. Therefore, $$a$$ and $$b $$ are irrelevant for the unreliability.

#### Proof of Theorem 36

Fix a $$\vec {\tau }'' \in {\mathbb {B}}^{\operatorname {CBE}_{T''}}$$, and let $${\tilde{v}}$$ be the node in $$T''$$ that replaced $$v $$ (i.e., that has the function of $$\operatorname {R}_{T'}$$). To prove the theorem, we need to show that

9 $$\begin{aligned}&\langle U(T)\rangle [\mathsf{L}_{v} \mapsto \langle U(T')\rangle ][\forall x \in \operatorname {CBE}_{T''}:\mathsf{L}_{x} \mapsto \tau ''_x] \nonumber \\&\quad = U(T'')(\vec {\tau }''). \end{aligned}$$

To prove this, note that we have

$$\begin{aligned}&\langle U_T\rangle [\mathsf{L}_{v} \mapsto \langle U(T')\rangle ][\forall x \in \operatorname {CBE}_{T''}\colon\mathsf{L}_{x} \mapsto \tau ''_x]\\&\quad = \Big (\langle U_T\rangle [\mathsf{L}_{v} \mapsto 1]\cdot \langle U_{T'}\rangle +\langle U_{T}\rangle [\mathsf{L}_{v} \mapsto 0] \\ &\qquad \cdot (1-\langle U_{T'}\rangle )\Big )[\forall x \in \operatorname {CBE}_{T''}\colon\mathsf{L}_{x} \mapsto \tau ''_x]\\&\quad = \langle U_T\rangle [\mathsf{L}_{v} \mapsto 1,\forall x \in \operatorname {CBE}_{T''}\colon\mathsf{L}_{x} \mapsto \tau ''_x] \\ &\qquad \cdot \langle U_{T'}\rangle [\forall x \in \operatorname {CBE}_{T''}\colon\mathsf{L}_{x} \mapsto \tau ''_x]\\&\qquad + \langle U_T\rangle [\mathsf{L}_{v} \mapsto 0,\forall x \in \operatorname {CBE}_{T''}\colon\mathsf{L}_{x} \mapsto \tau ''_x] \\ &\qquad \cdot (1-\langle U_{T'}\rangle [\forall x \in \operatorname {CBE}_{T''}\colon\mathsf{L}_{x} \mapsto \tau ''_x]). \end{aligned}$$

Here we used Lemmas 18 and 20, as well as the fact that $(\tau ''_x)^2 = \tau ''_x$ for all *x*. Now define $\vec {\tau }^0,\vec {\tau }^1 \in {\mathbb {B}}^{\operatorname {CBE}_{T}}$ and $\vec {\tau }' \in {\mathbb {B}}^{\operatorname {CBE}_{T'}}$ by

$$\begin{aligned} \tau ^0_x&= {\left\{ \begin{array}{ll} \tau ''_x, & \text { if } x \ne v ,\\ 0, & \text { if } x = v , \end{array}\right. }\\ \tau ^1_x&= {\left\{ \begin{array}{ll} \tau ''_x, & \text { if } x \ne v ,\\ 1, & \text { if } x = v , \end{array}\right. },\\ \tau '_x&= \tau ''_x. \end{aligned}$$

Note that this is well-defined since $\operatorname {CBE}_{T'} \subseteq \operatorname {CBE}_{T''}$ and $\operatorname {CBE}_{T} \subseteq \operatorname {CBE}_{T''} \cup \{v\}$. In this formulation we find that

10 $$\begin{aligned}&\langle U(T)\rangle [\mathsf{L}_{v} \mapsto \langle U(T')\rangle ][\forall x \in \operatorname {CBE}_{T''}\colon\mathsf{L}_{x} \mapsto \tau ''_x]\nonumber \\&\quad = \langle U_T\rangle [\forall x \in \operatorname {CBE}_{T}\colon\mathsf{L}_{x} \mapsto \tau ^1_x] \nonumber \\ &\qquad \cdot \langle U_{T'}\rangle [\forall x \in \operatorname {CBE}_{T'}\colon\mathsf{L}_{x} \mapsto c'_x] \nonumber \\&\qquad +\langle U_T\rangle [\forall x \in \operatorname {CBE}_{T}\colon\mathsf{L}_{x} \mapsto \tau ^0_x] \nonumber \\ &\qquad \cdot (1-\langle U_{T'}\rangle [\forall x \in \operatorname {CBE}_{T'}\colon\mathsf{L}_{x} \mapsto c'_x])\nonumber \\&\quad = U_T(\vec {\tau }^1)U_{T'}(\vec {\tau }')+U_T(\vec {\tau }^0)(1-U_T(\vec {\tau }')). \end{aligned}$$

Let $\vec {\Sigma }'' \in {\mathbb {B}}^{\operatorname {BE}_{T''}}$ be the random variable of Definition 32.2. By our assumption that $V \cap \operatorname {BE}_{T'} = \varnothing$, we know that $\operatorname {BE}_{T''}$ is the disjoint union of $\operatorname {BE}_{T}$ and $\operatorname {BE}_{T'}$. Thus we can write $\vec {\Sigma }'' = (\vec {\Sigma },\vec {\Sigma }')$, with $\vec {\Sigma }$ and $\vec {\Sigma }'$ independent random variables in ${\mathbb {B}}^{\operatorname {BE}_{T}}$ and ${\mathbb {B}}^{\operatorname {BE}_{T'}}$, respectively. Furthermore, all paths from $\operatorname {R}_{T}$ to elements of $\operatorname {BE}_{T}$ pass through ${\tilde{v}}$, and so the random variables $\vec {\Sigma }$ and $\operatorname {S}_{T''}({\tilde{v}},\vec {\Sigma }'',\vec {\tau }'')$ are independent. It follows that

11 $$\begin{aligned} U(T'')(\vec {\tau }'')\\&\!\!\!\!\!\!\!\!\!\!\!\!\!\!\!\!\!\!\!\!\!= \mathbb {P}(\operatorname {S}_{T}(\operatorname {R}_{T''},\vec {\Sigma }'',\vec {\tau }'') = \texttt{1}) \nonumber \\&\!\!\!\!\!\!\!\!\!\!\!\!\!\!\!\!\!\!\!\!\!= \mathbb {P}(\operatorname {S}_{T''}(\operatorname {R}_{T''},\vec {\Sigma }'',\vec {\tau }'') = \texttt{1}\mid \operatorname {S}_{T''}({\tilde{v}},\vec {\Sigma }'',\vec {\tau }'') = \texttt{1}) \nonumber \\&\!\!\!\!\!\!\!\!\!\!\!\cdot \mathbb {P}(\operatorname {S}_{T''}({\tilde{v}},\vec {\Sigma }'',\vec {\tau }'') = \texttt{1}) \nonumber \\&\!\!\!\!\!\!\!\!\!\!\!\!\!\!\!\! + \mathbb {P}(\operatorname {S}_{T''}(\operatorname {R}_{T''},\vec {\Sigma }'',\vec {\tau }'') = \texttt{1}\mid \operatorname {S}_{T''}({\tilde{v}},\vec {\Sigma }'',\vec {\tau }'') = \texttt{0}) \nonumber \\ &\!\!\!\!\!\!\!\!\!\!\!\cdot \mathbb {P}(\operatorname {S}_{T''}({\tilde{v}},\vec {\Sigma }'',\vec {\tau }'') = \texttt{0})\nonumber \\&\!\!\!\!\!\!\!\!\!\!\!\!\!\!\!\!\!\!\!\!\!= \mathbb {P}(\operatorname {S}_{T}(\operatorname {R}_{T},\vec {\Sigma },\vec {\tau }^1) = \texttt{1}) \nonumber \cdot \mathbb {P}(\operatorname {S}_{T'}(\operatorname {R}_{T'},\vec {\Sigma }',\vec {\tau }') = \texttt{1})\nonumber \\&\!\!\!\!\!\!\!\!\!\!\!\!\!\!\!\! + \mathbb {P}(\operatorname {S}_{T}(\operatorname {R}_{T},\vec {\Sigma },\vec {\tau }^0) = \texttt{1}) \nonumber \cdot \mathbb {P}(\operatorname {S}_{T'}(\operatorname {R}_{T'},\vec {\Sigma }',\vec {\tau }') = \texttt{0})\nonumber \\&\!\!\!\!\!\!\!\!\!\!\!\!\!\!\!\!\!\!\!\!\!= U_T(\vec {\tau }^1)U_{T'}(\vec {\tau }')+U_T(\vec {\tau }^0)(1-U_T(\vec {\tau }')). \end{aligned}$$

Combining (10) and (11), we have shown (9) and the proof is complete. $\square$

This theorem will be applied ‘in reverse’: given a large PCFT $T''$, one can calculate $U(T'')$ by finding a *quasimodule* $T'$ and its remainder $T$, and combining $U(T)$ and $U(T')$. In this sense, this theorem is analogous to *modular decomposition* of FTs [13], in which a FT’s unreliability is expressed in terms of that of its *modules*. Again, the key difference is that we allow not just modular decomposition, but also quasimodular decomposition.

**Fig. 6:**
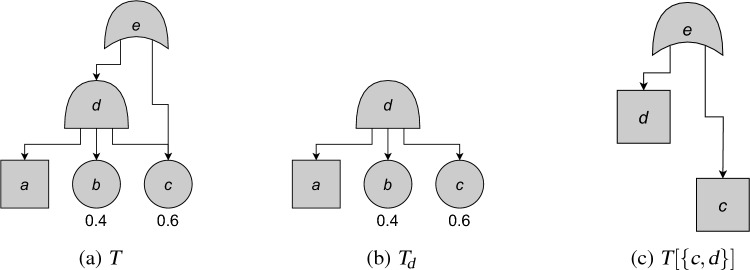
An example of the constructions of Definition 38. A PCFT $T$ is depicted in (a) (square nodes are CBEs). The sub-FT $T_d$ with root $d$ is depicted in (b). The FT $T[\{c,d\}]$ obtained by turning $c$, $d$ into CBEs is depicted in (c); note that this FT is also equal to $T[\{b,c,d\}]$, $T[\{a,c,d\}]$ and $T[\{a,b,c,d\}]$. From [3, Fig. 5]

## Proof of Correctness

In this section, we prove Theorem 29. The key idea is that we show, by induction, that each $g_v$ in Algorithm 1 equals $\langle U(T')\rangle$ for an appropriate sub-PCFT $T'$ of $T$. This is shown by using Theorem 36 to show that the substitution steps in line 17 correspond to quasimodular composition. In the end, we find that $g_{\operatorname {R}_{T}}$ corresponds to $\langle U(T)\rangle$, proving the correctness.

For notational convenience, we define two ways to construct new (PC)FTs from a FT.

### Definition 38

Let $T = (V,E,\gamma ,\textrm{p})$ be a FT.

1. Let $v \in V$. Then $T_v = (V_v,E_v,\gamma _v,\textrm{p}_v)$ is the FT consisting of the descendants of *v*, with *v* as a root.
2. Let $I \subseteq V$. Then $T[I]$ is the PCFT obtained from $T$ via the following procedure:
  For each $v \in I$, set $\gamma (v) = \texttt{CBE}$;
  For each $v \in I$, remove all outgoing edges;
  Then $T[I]$ is the PCFT consisting of all nodes reachable from the root.

These constructions are depicted in Fig. 6.

For a node $v$, we write $g_{v,0}$ for the value of $g_v$ at it initialization in Algorithm 1, i.e., at line 6,9 or 11. Furthermore, if $\gamma (v) \ne \texttt{BE}$, let $w_1,\ldots ,w_n$ be the elements of $\mathcal {S}_v = \{w \in V \mid \operatorname {id}(w) = v\}$, in the order in which they are picked in line 15; then $g_{v,i}$ is the value of $g_v$ after *i* iterations of the loop 14–18. Let $g_{v,\infty }$ be the value of $g_v$ at the end of the loop in lines 14–18 of Algorithm 1; this is the value $g_{v}$ has when the algorithm ends, and is used to substitute $\mathsf{L}_{v}$ in line 17. Thus $g_{v,\infty } = g_{v,0}$ if $\gamma (v) = \texttt{BE}$, and $g_{v,\infty } = g_{v,n}$ otherwise, in the notation above. Then Theorem 29 follows from the following result:

### Theorem 39

For $$v \in V$$, define $$\mathcal {I}_v = \{w \in V $$$$ \mid w \prec v \prec \operatorname {id}(w)\}$$.

1. One has $$g_{v,\infty } = \langle U(T_v[\mathcal {I}_v])\rangle $$.
2. Suppose $\gamma (v) \ne \texttt{BE}$ and let $w_1,\ldots ,w_n$ be the elements of $\mathcal {S}_v$, in the order in which they are picked in line 15. Then $g_{v,i} = \langle U(T_v[\mathcal {I}_v \cup \{w_{i+1},\ldots ,w_n\}])\rangle$ for $i \ge 0$.

Since $\mathcal{I}_{\operatorname {R}_{T}} = \varnothing$ and $T_{\operatorname {R}_{T}} = T$, Theorem 29 follows from point 1. by taking $v = \operatorname {R}_{T}$.

### Proof

We prove this by induction on $v$, and within a given $v$ by induction on $i$. If $\gamma (v) = \texttt{BE}$, then $T_v$ consists of a single BAS with failure probability $\textrm{p}(v)$; as such $U(T_v) = \textrm{p}(v) = g_v$. This proves the first statement for BEs.

Now suppose $\gamma (v) = \texttt{OR}$; the case that $\gamma (v) = \texttt{AND}$ is completely analogous. We will prove statement 2, as statement 1 is just the special case $i = n$. We start with the case $i=0$. Since $\mathcal {S}_v \cup \mathcal {I}_v = \{w \in V \mid w \prec v \preceq \operatorname {id}(w)\}$ and $v \preceq \operatorname {id}(w)$ for every child *w* of *v*, we have $\operatorname {ch}(v) \subseteq \mathcal {I}_v \cup \{w_1,\ldots ,w_n\}$. It follows that $T':= T_v[\mathcal {I}_v \cup \{w_{i+1},\ldots ,w_n\}]$ is a PCFT consisting of *v*, and all its children labeled $\texttt{CBE}$. Hence $U(T')(\vec {\tau }) = \max _{w \in \operatorname {ch}(v)} \tau _w$ for $\vec {\tau } \in {\mathbb {B}}^{\operatorname {ch}(v)}$, which is represented by the polynomial $1-\prod _{w \in \operatorname {ch}(v)} (1-\mathsf{L}_{w})$. This proves the statement for $i = 0$.

Now suppose the statement is true for a given $i-1$, and let $T_1 = T_v[\mathcal {I} \cup \{w_{i},\ldots ,w_n\}]$ and $T_2 = T_v[\mathcal {I} \cup \{w_{i+1},\ldots ,w_n\}]$. Then $T_2$ is obtained by replacing the CBE $w_i$ in $T_1$ by the PCFT $T_3 = T_{w_i}[(\mathcal {I}_v \cup \{w_{i+1},\ldots ,w_n\}) \cap \operatorname {desc}(w_i)]$, where $\operatorname {desc}(w_i)$ denotes the set of descendants of $w_i$. Note that since we pick the $w_j$ in a reverse topological order, all $w_j$ that are descendants of $w_i$ satisfy $j > i$; hence

$$ (\mathcal {I}_v \cup \{w_{i+1},\ldots ,w_n\}) \cap \operatorname {desc}(w_i) = (\mathcal {I}_v \cup \mathcal {S}_v) \cap \operatorname {desc}(w_i). $$

We now prove that this set is equal to $\mathcal {I}_{w_i}$. If $w \in (\mathcal {I}_v \cup \mathcal {S}_v) \cap \operatorname {desc}(w_i)$, then $w \prec w_i$ and $w_i \prec v \preceq \operatorname {id}(w_i)$; hence $w \in \mathcal {I}_{w_i}$, proving one inclusion. Conversely, if $w \in \mathcal {I}_{w_i}$, then $w_i \prec \operatorname {id}(w)$; hence $\operatorname {id}(w_i) \preceq \operatorname {id}(w)$ by Lemma 12. Hence $v \preceq \operatorname {id}(w_i) \preceq \operatorname {id}(w)$ and $w \in (\mathcal {I}_v \cup \mathcal {S}_v) \cap \operatorname {desc}(w_i)$, proving the other conclusion. We conclude that $T_3 = T_{w_i}[\mathcal {I}_{w_i}]$.

Now suppose that $T_3$ and $T_1$ share a BE *w*. If this were the case, then there is a path $v \rightarrow w$ not through $w_i$, namely any such path in $T_2$. Since $w \preceq w_i$ we conclude that $w_i \prec \operatorname {id}(w)$. But then $w \in \mathcal {I}_{w_i}$; since $T_3 = T_{w_i}[\mathcal {I}_{w_i}]$ this means that $\gamma (w) = \texttt{CBE}$ rather than $\texttt{BE}$, which is a contradiction. We conclude that no such *w* exist. Hence we can invoke Lemma 36 and conclude that

$$\begin{aligned} g_{v,i}&= g_{v,i-1}[\mathsf{L}_{w_i} \mapsto g_{w,\infty }] \\ &= \langle U(T_1)\rangle [\mathsf{L}_{w_i} \mapsto \langle U(T_3)\rangle ] = \langle U(T_2)\rangle , \end{aligned}$$

as was to be shown. $\square$

## Complexity

**Algorithm 2:**
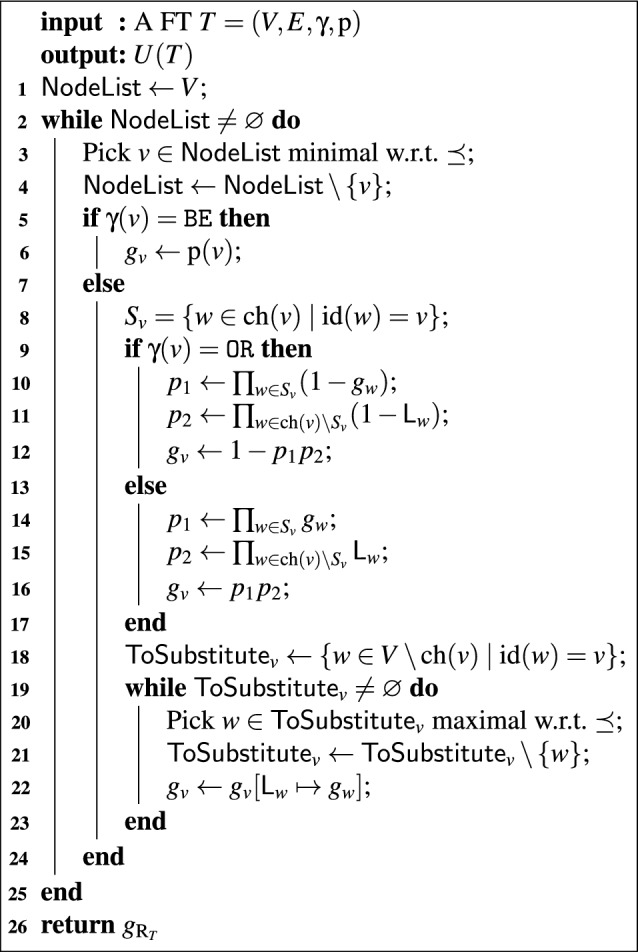
The algorithm $$\mathtt{SFPA2}(T)$$.

The complexity of Algorithm 1 can be bound in terms of graph parameters of the directed acyclic graph $T$. To do so, we first note that we can slightly rephrase the algorithm as follows. If $w$ is a child of $v$ and $w$ has only one parent, then $w$ is a maximal element of $\mathsf{ToSubstitute}_v$. As such, in line 15 these $w$ will be picked first. Therefore, we may as well do this replacement in lines 9 and 11 directly. This is incorporated in Algorithm 2, which has the same functionality as Algorithm 1 and therefore calculates $U(T)$ correctly. Note that the condition that *w* has only one parent is equivalent to $\operatorname {id}(w) = v$, which leads to our definition of $S_v$ in line 8.

Like the standard algorithm for reliability analysis for treelike FTs [4], Algorithm 2 works bottom-up. However, it is more complicated due to the fact that our main objects of interest are squarefree polynomials rather than real numbers, and operating on these induces a larger complexity. To describe this complexity, we introduce the following notation:

$$ X = \{v \in V \mid v \text { has multiple parents}\}. $$

Since in Algorithm 2 line 8, the set $S_v$ consists of all children of *v* with a single parent, the only $\mathsf{L}_{x}$ that are introduced satisfy $x \in X$. Thus each $g_v$ is an element of $\mathcal {A}(X)$, and as such has at most $2^{|X|}$ terms. Multiplying two such polynomials has complexity $\mathcal {O}(4^{|X|})$. Since substitution is just multiplication by Lemma 18, substitution has the same complexity. Next, we count the number of multiplications and substitutions. The FT $T$ has $|X|$ nodes with more than 1 parent and 1 node with 0 parents, so in total $|V|-|X|-1$ nodes have exactly one parent and are used in multiplications in lines 10 and 14 of Algorithm 2; in particular, there are at most $|V|$ multiplications. Furthermore, $|\mathsf{ToSubstitute}_v| \le |X|$ for each $v$, hence at each $v$ there are at most $|X|$substitutions in line 22. We conclude:

### Theorem 40

*Let* *X* *be as above. Then Algorithm* 2 *has time complexity* $\mathcal {O}(|V|(|X|+1){4}^{|X|})$.

For example, consider $X = \varnothing$; then *T* is treelike. In this case, Theorem 40 tells us that Algorithm 2 has time complexity $\mathcal {O}(|V|)$. Indeed, in this case $S_v = \operatorname {ch}(v)$ for all $v$, and each $g_v$ is a real number. Hence Algorithm 2 reduces to the standard bottom-up algorithm for treelike FTs, which is known to have linear time complexity [4]. In fact, Theorem 40 generalizes this result: it shows that for bounded $|X|$, time complexity of Algorithm 2 is linear. This makes Algorithm 2 a useful tool if the non-tree topology of an FT is concentrated in only a few nodes.

The complexity bound of Theorem 40 can be sharpened by realizing that a variable $\mathsf{L}_{w}$ only occurs in the computation of $g_v$ if $w \prec v \preceq \operatorname {id}(w)$. Using this as a bound we get the following result:

### Theorem 41

*Define*

$$ c = \max _{v \in V} |\{w \in X \mid w \prec v \preceq \operatorname {id}(w)\}|. $$

*Then Algorithm* 2 *has time complexity* $\mathcal {O}(|V|(c+{1}){4}^c)$.

## Experiments

**Table 1:** Statistics of the industry FT benchmarks, and computation time (in seconds) and memory usage (in KB) of FT reliability algorithms

	Aralia		FFORT		Industry		Railway		Sprinkler	
FTs	40		20		3		8		3	
Nodes	61–3189		10–69		57–251		100–330		66	
Edges	72–4880		9–73		58–250		131–605		73	
MP nodes	0.6–50.7%		0–23.4%		0–3.5%		32–50%		4.5%	

	Time	Memory	Time	Memory	Time	Memory	Time	Memory	Time	Memory
*SFPA*										
Min	0.56	**1271**	**0**.**01**	**122**	0.14	**845**	–	15,063	0.56	**1050**
Median	–	44,124	**0**.**03**	**244**	0.18	**1009**	–	19,742	0.62	**1129**
Max	–	***157,718***	0.44	**1101**	0.68	**1833**	–	93,160	0.69	**1437**
Overtime	78%		0%		0%		100%		0%	
*Coyan*										
Min	**0**.**01**	3968	0.03	13,814	**0**.**05**	13,312	**0**.**05**	**13,312**	**0**.**04**	13,312
Median	**0**.**07**	**14,080**	0.05	13,184	**0**.**06**	13,312	**0**.**07**	**13,504**	**0**.**04**	13,312
Max	–	168,704	**0**.**09**	13,568	**0**.**10**	13,824	**0**.**35**	**18,560**	**0**.**07**	13,312
Overtime	**10%**		0%		0%		0%	0%		
*STORM*										
Min	0.57	39,296	0.19	38,388	1.93	39,552	1.33	38,912	1.97	41,216
Median	22.96	222,464	1.50	38,636	3.08	42,368	3.05	47,296	2.30	41,216
Max	–	3,111,196	3.05	42,112	5.72	60,032	9.04	120,960	3.29	41,472
Overtime	43%		0%		0%		0%		0%	

Timeout is 60 s; min, median, max are not reported when these went overtime. Bold values are best-performing algorithms for that benchmark; bold italic means that this includes timeout FTs, so the comparison is not entirely fair

**Table 2:** Statistics of the industry FT benchmarks, and computation time (in seconds) and memory usage (in KB) of FT reliability algorithms

	Scram1		Scram2		Scram3		Cogen	
FTs	128		128		35		35	
Nodes	220–272		161–1633		66–10,142		100–10,000	
Edges	229–354		167–1752		73–18,493		260–28,050	
MP nodes	2.9–4.9%		0.2–3.1%		5.6–10.9%		52.0–58.4%	

	Time	Memory	Time	Memory	Time	Memory	Time	Memory
*SFPA*								
Min	0.43	**1606**	0.26	**1141**	0.41	**2159**	1.80	**2339**
Median	6.64	**7294**	1.24	**1966**	–	44,143	–	18,268
Max	–	93,533	15.25	**10,634**	–	***92,428***	–	37,555
Overtime	7.0%		**0%**		83%		66%	
*Coyan*								
Min	**0**.**01**	13,568	**0**.**01**	13,440	**0**.**05**	4864	**0**.**03**	13,312
Median	**0**.**02**	13,696	**0**.**02**	13,696	–	***13,312***	**0**.**25**	**14,208**
Max	**0**.**48**	**14,336**	**0**.**06**	15,474	–	262,052	**3**.**51**	**31,400**
Overtime	**0%**		**0%**		**51%**		**0%**	
*STORM*								
Min	0.50	48,000	0.36	43,648	2.40	41,600	–	41,728
Median	4.14	74,880	3.91	64,960	–	317,184	–	102,528
Max	12.01	213,760	–	766,208	–	64,131,488	–	5,302,680
Overtime	**0%**		1%		83%		100%	

Timeout is 60 s; min, median, max are not reported when these went overtime. Bold values are best-performing algorithms for that benchmark; bold italic means that this includes timeout FTs, so the comparison is not entirely fair

**Fig. 7:**
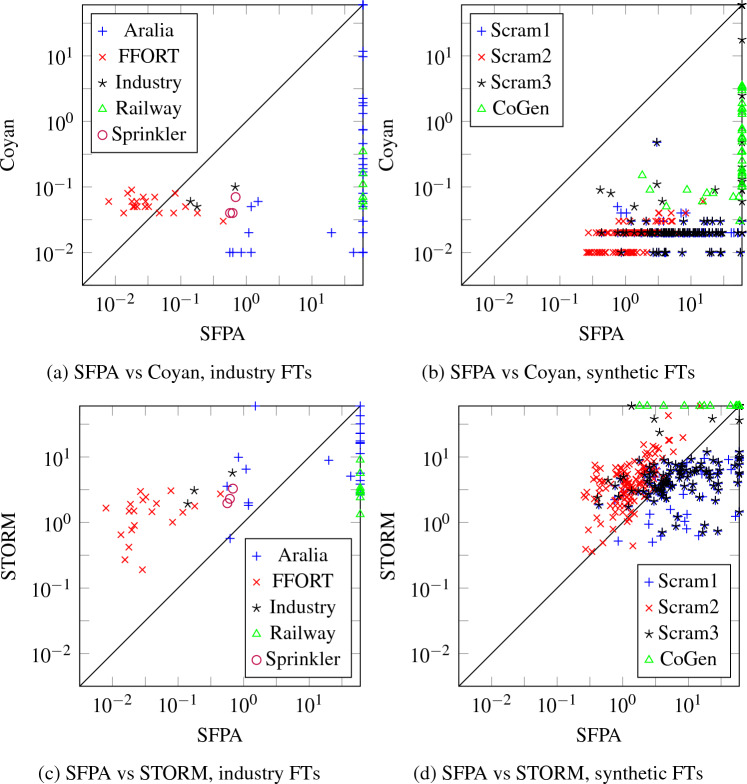
Time comparison between SFPA, Coyan and STORM (in seconds). Timeout is 60 s

We evaluate $\texttt{SFPA}$’s performance against two state-of-the-art tools: STORM [5], which computes FT reliability using a BDD-based approach plus modularization; and Coyan [6], which translates FT reliability to a weighted model count (WMC) problem and employs state-of-the-art WMC solvers. All three approaches have worst-case exponential runtime. All experiments are performed on a Ubuntu virtual machine with 6 cores and 8GB ram, running on a PC with an Intel Core i7-10750HQ 2.8GHz processor and 16GB memory; the artifact is available at [7].

We consider a number of benchmarks, consisting of both expert-created and synthetic fault trees. Our industry FT benchmarks are:

1. A number of 54 FTs from [5], divided into the subsets *Aralia*, *Industry*, *Railway* and *Sprinkler*.
2. A benchmark of 20 FTs presented originally in [42] (*FFORT*).

Since these FTs are limited in number, we also consider a number of randomly generated benchmarks:

1. A collection of 128 FTs, generated using SCRAM [15], used as a benchmark set in [5] (*Scram1*)
2. A collection of 128 FTs, generated for this paper using SCRAM. These FTs are specifically generated to have less nodes with multiple parents, to test the theoretical results of Section 10 (*Scram2*).
3. A collection of 35 FTs, generated using SCRAM, used as a benchmark set in [6]. These were created to vary in size considerably (*Scram3*).
4. A collection of 35 FTs, generated using a novel algorithm presented in [6]. The generation algorithm was developed because SCRAM-generated FTs often have a small number of BEs with a large impact on the top-level event, which is usually not the case in real-life FTs [6] (*Cogen*).

**Fig. 8:**
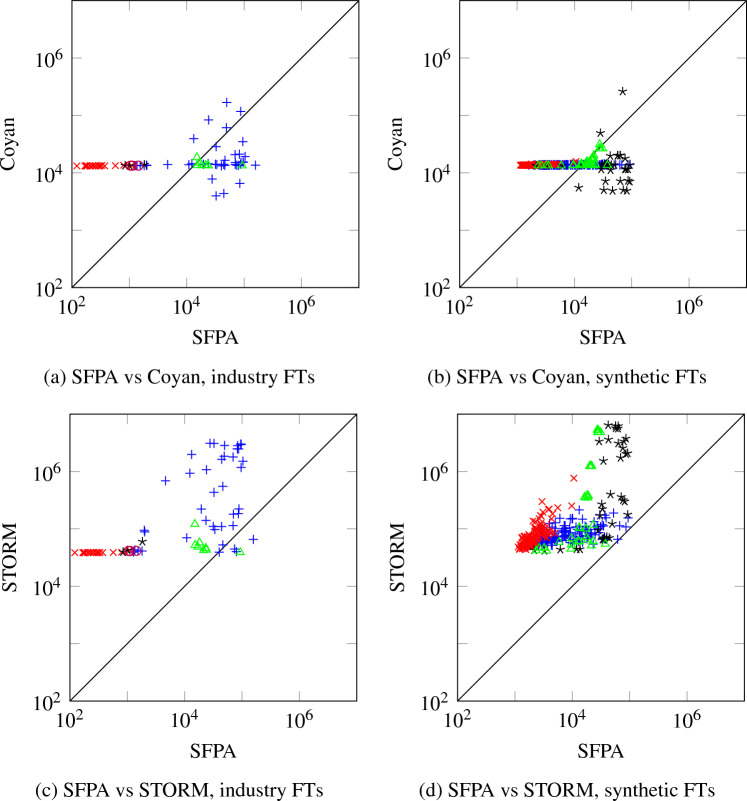
Memory comparison between SFPA, Coyan and STORM (in KB). Timeout is 60 s

**Fig. 9:**
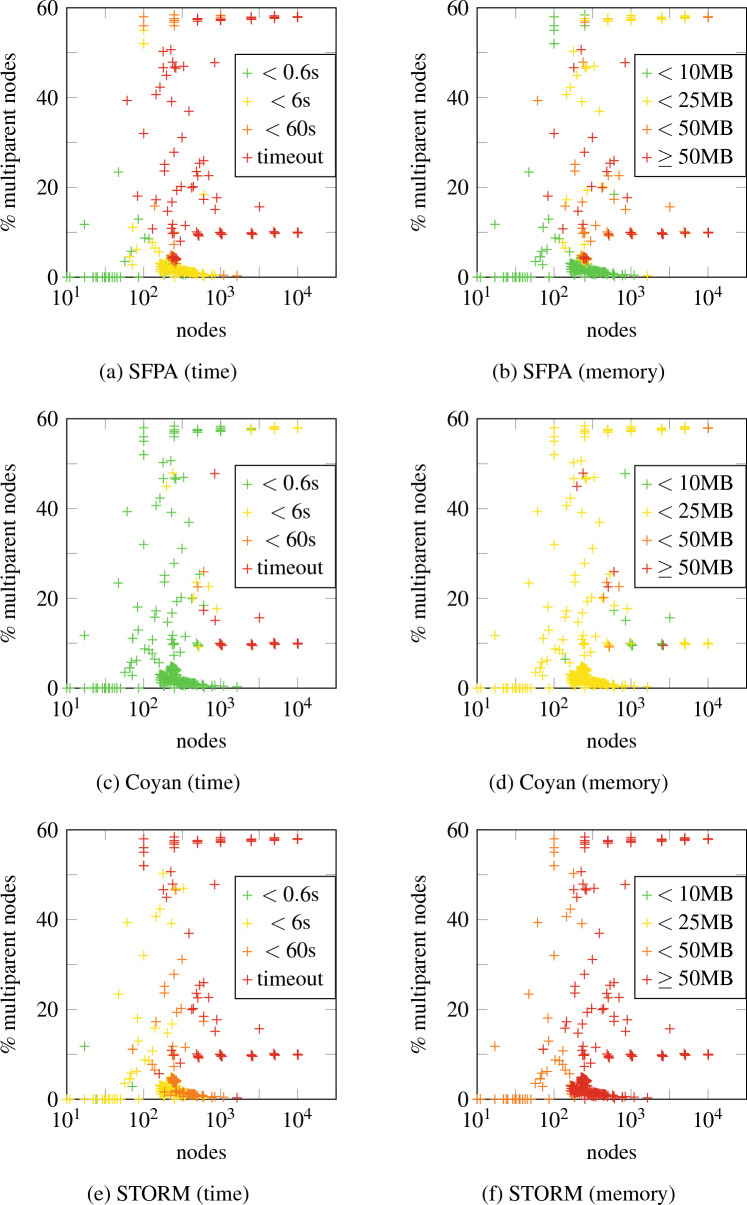
Time and memory performance of SFPA, Coyan and STORM, on all our benchmark FTs, arranged by number of nodes and percentage of multiparent nodes. Timeout is 60 s

**Fig. 10:**
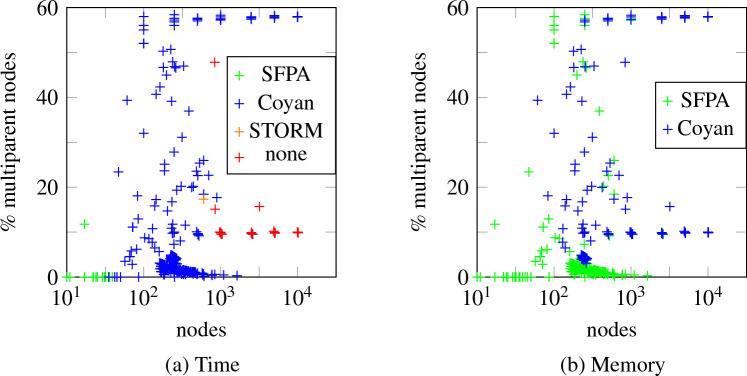
Time and memory performance of SFPA, Coyan and STORM on all benchmarks. For each FT, the fastest/most memory efficient algorithm is shown; none means that all algorithms timed out

On each FT, we compute the reliability using SFPA, Coyan and STORM, and compare the performance in terms of computation time and memory usage. Timeout is set at 60 s. The results are in Tables 1 and 2. The results are also presented as direct comparisons in Figs. 7 and 8, and the impact of number of nodes and percentage of multiparent nodes is presented in Fig. 9. In Fig. 10 we depict which algorithm performs the best on each FT, in terms of time and memory.

### Time

The results clearly show that in most instances, Coyan outperforms SFPA timewise: except for some of the FTs in the FFORT benchmark, which consists of small FTs ($\le 69$ nodes), Coyan is faster than SFPA by quite a margin. In fact, on 87% of all FTs, Coyan is at least 10$\times$ as fast as SFPA. To an extent, this can probably be attributed to the fact that Coyan is written in Rust and invokes state-of-the-art model counting tools, while SFPA only has a proof-of-concept Python implementation.

The picture between SFPA and STORM is more nuanced: on the industry FTs SFPA is generally faster for smaller FTs (FFORT, Industry, Sprinkler), but slower for large FTs (Aralia, Railway). On randomly generated FTs the two methods perform about evenly, with SFPA performing better on the Scram2 benchmark. This reflects the results of “Complexity”, where we showed that the number of multiparent nodes exponentially impacts computation time of SFPA. By contrast, for a BDD-based approach the presence of *any* multiparent nodes means one cannot use the bottom-up algorithm and has to rely on creating the BDD, which is usually slower than the bottom-up approach. Furthermore, modularization may only be of limited use depending on the position of the multiparent nodes. The impact of multiparent nodes on the time performance of SFPA can also clearly be seen in Fig. 9.

Overall though, it is clear that scalability is still an issue for SFPA, as it struggles to compute reliability on larger FTs (*Railway*, *Scram3*, *Cogen*). In Fig. 9, it can be seen that SFPA has timeouts for many FTS with $>100$ nodes, and that this is especially prevalent for FTs with $>10\%$ multiparent nodes

### Memory

Memorywise, STORM performs worst: with the exception of a few industry FTs, SFPA and Coyan both have consistently better memory performance. With regards to the difference between SFPA and Coyan, the clearest story emerges from Fig. 9: for FTs with $<100$ nodes, SFPA performs better; for FTs with $>100$ and $<1000$ nodes, Coyan performs better on FTs with many multiparent nodes, and SFPA performs better on FTs with fewer multiparent nodes; and for FTs with $>1000$ nodes, Coyan performs better. Again, this shows the impact of multiparent nodes on SFPA. An important sidenote here is that our benchmarks do not contain FTs with $<5\%$ multiparent nodes and $>1000$ nodes; it would be interesting to see how SFPA performs on such FTs memory-wise.

## Conclusion

This paper introduces SFPA, a novel algorithm for calculating fault tree unreliability based on squarefree polynomial algebras. We have developed the algebraic framework necessary to prove its validity and given complexity bounds in terms of the number of multiparent nodes.

Our experiments on industry and synthetic FTs show that timewise, SFPA is competitive with state-of-the-art BDD-based STORM model checker [5], though the novel WMC-based tool Coyan [6] outperforms SFPA by a factor 10 or more. Furthermore, analyzing FTs with $>500$ nodes with SFPA is computationally unfeasible. In memory usage, SFPA clearly outperforms STORM and is competitive with Coyan, especially on FTs with fewer nodes and fewer multiparent nodes. Thus, in general, Coyan is to be preferred for large, complicated FTs; SFPA is to be preferred for smaller FTs; and for medium-size FTs with few multiparent nodes, the choice between SFPA and Coyan is a tradeoff between computation time and memory usage. Regardless of the experimental results, though, SFPA is based on completely different algebraic foundations compared to STORM and Coyan, and offers a valuable new perspective on fault tree reliability.

There are several directions for future work. First, our proof-of-concept Python implementation of SFPA can undoubtedly be improved to reduce computation time. The underlying theory could be expanded to increase efficiency as well. For example, a new formal variable $\mathsf{U}_v$ for each $1-\mathsf{L}_{v}$ could be introduced to decrease the number of terms in the expression of $g_v$, when $v$ is an $\texttt{OR}$-gate, from $2^{|\operatorname {ch}(v)|}-1$ to 2. This has the potential to reduce computation time. In this case, new computation rules such as $\mathsf{L}_{v}\mathsf{U}_v = 1$ need to be introduced.

Second, it would be worthwhile to compare FT unreliability computation algorithms with regards to their numerical stability. SFPA itself is based on polynomial operations, which come down to addition and multiplication on real numbers; however, these polynomials can be of exponential size, so rounding errors could potentially accumulate. The substitution $\mathsf{U}_v = 1-\mathsf{L}_{v}$ above reduces polynomial size, so it can also help in improving numberical stability.

Third, our proof of validity is involved and requires quite some underlying theory. This would make it a good candidate to formalize in a theorem-proving environment, perhaps extending existing work on fault tree formalization [43], to have even stronger guarantees that the computation is correct.

Finally SFPA-like methods have the potential to be applied to other problems in risk analysis. A reasonable target is (static) attack tree analysis: instead of using polynomial algebras over the reals, this would entail polynomial semirings over the semirings used to model attack tree metrics [28]. A more challenging application would be to encode the order-related behaviour of dynamic fault trees and attack trees algebraically.

## Data Availability

An artifact of the experiments, containing Python code, the analyzed fault trees, and the results, can be found at [7].

## Footnotes

1 As we will see later, all squarefree polynomials that will appear in our algorithm represent probabilities in some way, and will satisfy $\alpha _Y \in [0,1]$ for all *Y*. Nevertheless, in our definition of $\mathcal {A}(X)$, we allow for any real number, as this makes formulations more straightforward.
